# Evolutionary Basis of High-Frequency Hearing in the Cochleae of Echolocators Revealed by Comparative Genomics

**DOI:** 10.1093/gbe/evz250

**Published:** 2019-11-15

**Authors:** Hui Wang, Hanbo Zhao, Keping Sun, Xiaobin Huang, Longru Jin, Jiang Feng

**Affiliations:** 1 Jilin Provincial Key Laboratory of Animal Resource Conservation and Utilization, Northeast Normal University, Changchun, China; 2 College of Life Science, Jilin Agricultural University, Changchun, China; 3 Vector Laboratory for Zoonosis Control and Prevention, Dali University, China

**Keywords:** adaptive evolution, cochlea, comparative genomics, echolocation

## Abstract

High-frequency hearing is important for the survival of both echolocating bats and whales, but our understanding of its genetic basis is scattered and segmented. In this study, we combined RNA-Seq and comparative genomic analyses to obtain insights into the comprehensive gene expression profile of the cochlea and the adaptive evolution of hearing-related genes. A total of 144 genes were found to have been under positive selection in various species of echolocating bats and toothed whales, 34 of which were identified to be related to hearing behavior or auditory processes. Subsequently, multiple physiological processes associated with those genes were found to have adaptively evolved in echolocating bats and toothed whales, including cochlear bony development, antioxidant activity, ion balance, and homeostatic processes, along with signal transduction. In addition, abundant convergent/parallel genes and sites were detected between different pairs of echolocator species; however, no specific hearing-related physiological pathways were enriched by them and almost all of the convergent/parallel signals were selectively neutral, as previously reported. Notably, two adaptive parallel evolved sites in TECPR2 were shown to have been under positive selection, indicating their functional importance for the evolution of echolocation and high-frequency hearing in laryngeal echolocating bats. This study deepens our understanding of the genetic bases underlying high-frequency hearing in the cochlea of echolocating bats and toothed whales.

## Introduction

Dawkins used bat echolocation to illustrate features of “good design” through evolution via natural selection in “The Blind Watchmaker” (Dawkins 1986). Echolocation is a complex sensory system that is usually used for navigation, localization, and hunting in environments where visibility is limited (Jones and Teeling 2006; Au and Simmons 2007). Echolocation evolved independently in bats and toothed whales and has been used as a remarkable example of convergent evolution derived from similar selective pressures, associated with the night sky for bats and the dark conditions underwater for whales (Li et al. 2010; Liu et al. 2010; Thomas and Hahn 2015). For both evolutionary biologists and ecologists, the molecular mechanisms behind the well-developed echolocation in bats and whales have been an intriguing topic.

High-frequency hearing is an important component of echolocation and is essential for echolocators to perceive ultrasonic signals (Madsen et al. 2004; Li et al. 2008; Churchill et al. 2016). Both echolocating bats and toothed whales have evolved remarkable high-frequency hearing ability accompanying echolocation. The cochlea is a crucial component of the auditory system, playing important roles in sound perception, signal processing, and transmission to the brain (Dallos and Fakler 2002; Salorio-Corbetto et al. 2017). Some morphological and physiological studies have demonstrated the presence of specific structures and physiological activities suitable for the perception of ultrasonic signals in the cochleae of different bat species (Neuweiler 1989, 1990). It was recently reported that several high-frequency hearing-related genes, such as *Prestin* and *KCNQ4*, had been under positive selection (Li et al. 2008; Liu et al. 2011; Shen et al. 2012). These genes were also shown to have undergone sequence convergence between lineages of echolocating bats and toothed whales. This suggests that convergent molecular adaptation associated with high-frequency hearing has occurred alongside the evolution of echolocation (Li et al. 2008; Liu et al. 2010; Davies et al. 2012).

However, there have been few large-scale analyses on adaptive evolution among all genes expressed in the cochleae of echolocators. In particular, there is limited understanding of molecular convergence/parallel signals at the genomic level among different types of echolocator. Hence, this study was established to perform a comprehensive analysis of all genes expressed in the cochleae of echolocating bats and whales, in comparison with those in other nonecholocating mammals, which could offer key insights into the origin and evolution of high-frequency hearing and echolocation. Recent progress in sequencing technologies and bioinformatics provides appropriate tools for conducting this study. These tools include approaches for performing comparative transcriptomic analysis, which have had a considerable impact on evolutionary biology and facilitated investigation of the genetic basis of adaptive evolution (Hu et al. 2017; Gayk et al. 2018).

In this study, we applied comparative genomic analyses to uncover the evolutionary forces that have shaped the trajectory of genes responsible for high-frequency hearing in various echolocators. All echolocating bats and whales analyzed in this study can be roughly divided into four types according to their phylogenetic relationships, vocal organs, and acoustic structures: constant-frequency (CF) bats, frequency-modulated (FM) bats, tongue-click bats, and echolocating toothed whales. Cochleae from the three types of echolocating bat (CF, FM, and click bats) were collected and used for RNA-Seq to clarify the genes expressed in this anatomical region. Then, our sequenced transcriptomic gene sets were combined with the genomes of 16 other echolocating or nonecholocating mammals to perform evolutionary analyses. The main aims of this work are as follows: 1) to comprehensively investigate the positively selected genes involved in hearing in echolocating bats and toothed whales; 2) to detect to what extent convergent/parallel evolution has occurred between different echolocating bats and whales; and 3) to obtain insights into the interactions of adaptively evolved hearing-related genes in hearing-related pathways and physiological processes.

## Materials and Methods

### Ethics Statement

In accordance with the regulations of Wildlife Conservation of the People’s Republic of China (Chairman Decree [2004] No. 24), permits are required only for species included in the list of state-protected and region-protected wildlife species. None of the bat species used in this study is an endangered or region-protected animal, so no specific permission was required. All animal experimental procedures were approved by the National Animal Research Authority of Northeast Normal University, China (approval number: Nenu-20080416), and the Forestry Bureau of Jilin Province, China (approval number: [2006] 178).

### Sample Collection

During July 2016, the cochleae of echolocating bats, namely, *Aselliscus stoliczkanus* (CF bat), *Taphozous melanopogon* (FM bat), and *Rousettus leschenaultii* (click bat) were collected in Yunnan Province, China. Three adult females of each bat species were separately selected for RNA-Seq. A pair of cochlea tissues from each individual was collected, flash frozen in liquid nitrogen, and then placed in a −80 °C freezer until processed for total RNA isolation.

### RNA Extraction and cDNA Library Construction

Total RNA was isolated from each sample using TRIzol reagent (Life Technologies Corp., Carlsbad, CA), in accordance with the manufacturer’s protocol. The quantity and quality of total RNA were measured using an Agilent 2100 bioanalyzer (Agilent Technologies, Palo Alto, CA) and gel electrophoresis. Equal amounts of RNA were used during the conversion of mRNA into cDNA. Then, three paired-end cDNA libraries of each bat species were generated using mRNA-Seq assay for transcriptome sequencing on the Illumina Hiseq 4000 platform. Short sequence reads of 150 bp were generated. Raw sequence data were deposited into the NCBI Sequence Read Archive database (SRA run accession numbers: *A. stoliczkanus*: SRS3011421, *T. melanopogon*: SRS3011514, *R. leschenaultii*: SRS3011407).

### Transcriptome Assembly and Functional Annotation

The raw reads were filtered by removing the following: reads with adaptors; reads with unknown “N”; and low-quality reads containing >50% low-quality bases (*Q* value ≤20). Because there was no reference genome for the three bat species, de novo sequence assembly was carried out separately for each bat species using Trinity v.2.4.0 (Grabherr et al. 2011) with the default parameters. The assembled contigs with a minimum length of 200 bp were used for further analyses. After transcriptome assembly, CD-Hit v.4.6.6 (Li and Godzik 2006) was used to reduce sequence redundancy of the transcriptome with the default parameters. All remaining contigs are described as unigenes in the following text.

Basic annotations of unigenes included protein functional annotation, KOG functional annotation, Gene Ontology (GO) annotation, and Kyoto Encyclopedia of Genes and Genomes (KEGG) pathway annotation. In detail, we used BlastX (http://www.ncbi.nlm.nih.gov/BLAST/) with an E-value threshold of 1e − 5 in the NCBI nonredundant protein (Nr) database (http://www.ncbi.nlm.nih.gov/, last accessed May 15, 2018), the Swiss-Prot protein database (http://www.expasy.ch/sprot/, last accessed May 15, 2018), the KOG database (Tatusov et al. 2003), and the KEGG database (https://www.genome.jp/kegg/, release 87.0, July 1, 2018). The GO annotation and functional classification of unigenes were performed using Blast2GO v.4.0.7 (Conesa et al. 2005) and WEGO v.2.0 (Ye et al. 2006), respectively. Next, two programs, TransDecoder v.3.0.1 (http://transdecoder.github.io/) and MAKER v.2.31.10 (Cantarel et al. 2007), were applied to obtain the open reading frames (ORFs) of the unigenes. The remaining unigenes that could not be aligned to any protein database were scanned using ESTScan v.3.0 (Iseli et al. 1999), producing the predicted coding region and direction. Finally, after removing the CDSs that were shorter than 150 bp, all eligible CDSs of the unigenes were translated into amino acid (aa) sequences in accordance with the standard codon table.

### Identification and Alignment of Orthologous Genes

**Fig. 1.**
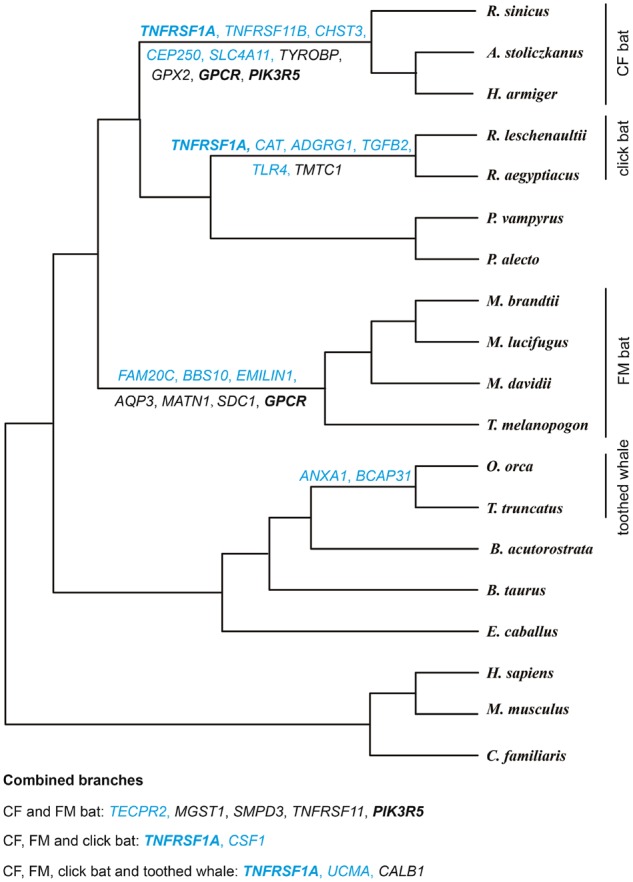
—Positively selected genes identified in the seven foreground branches. The PSGs with parallel sites are shown in blue. Bold typeface indicates genes under positive selection along more than two foreground branches.

The translated amino acid sequences of *A. stoliczkanus*, *T. melanopogon*, and *R. leschenaultii* were pooled together in a protein database with sequences (length >50 aa) from another 16 mammalian genomes, including those of 2 CF bats (*Rhinolophus sinicus* and *Hipposideros armiger*), 3 FM bats (*Myotis davidii*, *Myotisbrandtii*, and *Myotislucifugus*), 1 click bat (*Rousettus aegyptiacus*), 2 nonecholocating bats (*Pteropus vampyrus* and *Pteropus alecto*), 2 echolocating toothed whales (*Orcinus orca* and *Tursiops truncatus*), 1 nonecholocating baleen whale (*Balaenoptera acutorostrata*), and 5 other nonecholocating mammals (*Homo sapiens*, *Mus musculus*, *Bos taurus*, *Equus caballus*, and *Canis lupus familiaris*) (fig. 1).

Next, self-to-self BlastP was conducted for all amino acid sequences with an E-value cut-off of 1e − 5; hits with identity <30% and coverage <30% were removed. Orthologous groups were constructed from the BlastP results using OrthoMCL v.2.0.9 (Li et al. 2003) with the default settings. All of the identified orthologous groups were calculated and presented in a Venn diagram. Then, one-to-one single-copy orthologous genes were extracted by a Perl script. ORFs in each one-to-one orthologous set were aligned using PRANK v.140603 (Löytynoja and Goldman 2010) with the following parameters: −f = fasta -F -codon -noxml -notree -nopost. The alignment for each locus was trimmed by Gblocks v.0.91b (Castresana 2000) (parameters: −t = c, −b3 = 1, −b4 = 6, −b5 = n) to reduce the rate of false-positive predictions by filtering out sequencing errors, incorrect alignments, and nonorthologous regions based on codons.

### Positive Selection Analyses

The selective pressures were estimated using different codon substitution site models implemented in CodeML from phylogenetic analysis with maximum-likelihood (ML) software (PAML 4.8) (Yang 2007). By comparing ω = d*N*/d*S*, the ratio of nonsynonymous (d*N*) to synonymous (d*S*) substitutions, among sites and branches, the form and intensity of natural selection can be revealed, with ω < 1, ω  =  1, and ω > 1 indicating negative selection, neutral evolution, and positive selection, respectively. A well-established species tree based on previously reported phylogenetic studies was used in this analysis (fig. 1) (Jones and Teeling 2006; Mcgowen et al. 2009; Stoffberg et al. 2010; Mcgowen 2011; Chen et al. 2017).

A branch-site model (parameters: null hypothesis: model = 2, NSsites = 2, fix_omega = 1, omega = 1; alternative hypothesis: model = 2, NSsites = 2, fix_omega = 0, omega = 1) was used to identify positively selected genes (PSGs) in the targeted lineages, with the other lineages being specified as background branches (fig. 1). The targeted lineages, including CF bat, FM bat, click bat, and toothed whale, were in turn set as foreground branch. Besides, combined branches, including CF and FM branches; CF, FM, and click bat branches; and CF, FM, click bat, and toothed whale branches (fig. 1), were also separately used as foreground branches. A likelihood ratio test was established to compare a model that allows sites to be under positive selection (ω > 1) on the foreground branch with a null model in which sites may evolve neutrally (ω  =  1) and under purifying selection (ω  <  1) with a posterior probability in excess of 0.90 based on the Bayes empirical Bayes (BEB) results (Yang et al. 2005). At the same time, multiple testing was corrected by applying a false discovery rate (FDR) method implemented in R v.3.5.1 (http://www.r-project.org/) with an FDR-adjusted *P* value <0.05.

To determine the hearing-related genes among the PSGs, we searched for detailed information on them in GeneCards (https://www.genecards.org/), InterProScan (https://www.ebi.ac.uk/interpro/search/sequence-search), PubMed (https://www.ncbi.nlm.nih.gov/pubmed/), and published research papers based on their basic annotation information. Finally, we performed GO and KEGG functional enrichment analyses for each of seven groups of hearing-related PSGs and the combined group of the all PSGs using the GeneTrail2 method (Stockel et al. 2016). Significantly enriched GO terms and KEGG pathways were further subjected to a hypergeometric test to estimate significance (*P *<* *0.05).

### Identification of Parallel/Convergent Sites among Echolocating Mammals

To determine whether similar evolutionary patterns have occurred in animals who have developed the ability to echolocate and are habitually exposed to high-frequency sound but live in diverse environments, we searched for parallel/convergent amino acid substitutions from the internal nodes to terminal branches along paraphyletic lineages of species with the ability to hear high-frequency sounds. The parallel/convergent sites among each pairwise comparison were identified in accordance with previously described methods (Foote et al. 2015). Briefly, we reconstructed the ancestral amino acid sequences for all single-copy genes using the Bayesian approach (Rate Ancestor = 1) implemented in the BASEML program from the PAML package (Yang 2007). The two members of each pair of echolocators were compared, after which six pairwise comparisons were conducted: CF versus FM, CF versus click bat, CF versus toothed whale, FM versus click bat, FM versus toothed whale, and click bat versus toothed whale (Supplementary fig. 1, Supplementary Material online). For each of the six pairwise comparisons, the extant amino acid sequences at each position were compared with the ancestral sequence at the node corresponding to the most recent ancestor. We identified convergent/parallel amino acid sites using the following criteria: 1) the amino acid residues were identical at two extant compared nodes; and 2) the amino acid residues were different at the tested extant node and the tested most recent common ancestor. 3) Sites were identified as “convergent” if the amino acid residues differed at the two corresponding most recent common ancestors and as “parallel” if the amino acid residues were identical at the two corresponding most recent common ancestors. We used the software CONVERG 2 (Zhang and Kumar 1997) to test whether the observed convergent/parallel substitutions in focal branches had been fixed randomly or due to natural selection. We also focused on those genes for which convergent/parallel sites were identified in all tested echolocating mammals and performed GO and KEGG analyses on them.

In addition, to determine the background levels of parallel/convergent sites that occurred between echolocating species and nonecholocating species, we performed similar pairwise comparisons between corresponding echolocating species and nonecholocating species (Supplementary fig. 1, Supplementary Material online). Then, we compared the numbers of parallel/convergent genes and sites identified from every comparison between echolocating species with those identified from their equally phylogenetically distant controls. For example, we compared the number of parallel/convergent genes from CF versus click bat with the number from CF versus nonecholocating bat. Detailed information of the compared groups is listed in table 1. Here, the nonecholocating bats are *P. vampyrus* and *P. alecto* and the nonecholocating whale is *B. acutorostrata*. Subsequently, we evaluated the differences of the numbers of parallel genes from echolocating versus echolocating groups and echolocating versus nonecholocating groups by paired-sample *t*-test at a significance level of *P *<* *0.05. Then, we performed similar tests on the numbers of parallel sites, convergent genes, and convergent sites.

**Table 1:** Number of Parallel/Convergent Genes and Sites Identified among Pairwise Comparisons of Echolocating Species and Their Equally Phylogenetically Distant Controls

Comparisons between Echolocating Species vs. Echolocating Species			Comparisons between Echolocating Species vs. Nonecholocating Species		
	Num. of Parallel Gene (Site)	Num. of Convergent Gene (Site)		Num. of Parallel Gene (Site)	Num. of Convergent Gene (Site)
CF vs. FM	154 (171)	0	CF vs. nonecholocating bat	117 (124)	1 (1)
CF vs. click bat	207 (248)	3 (3)	CF vs. nonecholocating bat	117 (124)	1 (1)
FM vs. click bat	177 (203)	6 (6)	FM vs. nonecholocating bat	118 (122)	2 (2)
CF vs. toothed whale	119 (129)	11 (11)	CF vs. nonecholocating whale	84 (89)	5 (5)
FM vs. toothed whale	94 (98)	2 (2)	FM vs. nonecholocating whale	87 (92)	3 (3)
Click bat vs. toothed whale	96 (104)	11 (11)	Click bat vs. nonecholocating whale	90 (97)	3 (3)
Click bat vs. toothed whale	96 (104)	11 (11)	Nonecholocating bat vs. toothed whale	62 (65)	5 (5)

### Localization of Important Sites and Protein Network Analyses

To obtain insight into the functional significance of the putatively important sites, we mapped positively selected sites and parallel/convergent sites onto the proteins’ secondary and 3D structures. We identified the secondary structures of genes using InterProScan (http://www.uniprot.org/). We also reconstructed the 3D structures of proteins for the corresponding genes using SWISS-MODEL (https://swissmodel.expasy.org/). Then, we built the corresponding homologous 3D structures, and visualized and modified them using PyMOL v.2.1.1 (The PyMOL Molecular Graphics System, Schrödinger LLC; https://www.pymol.org/).

Furthermore, to reveal the relationships of those important hearing-related PSGs and convergent/parallel evolved genes, we searched for their associations and performed global protein network analyses using STRING v.10 (https://string-db.org/). In this way, we revealed the direct and indirect (functionally associated with no direct interaction) associations between those genes.

## Results

### Sequence Analysis

In this study, 34,524,892, 33,309,261, and 37,228,691 raw 150-bp pair end reads were generated for *A. stoliczkanus*, *T. melanopogon*, and *R. leschenaultii*, respectively (supplementary table S1, Supplementary Material online). After removing adapters and low-quality reads, 33,654,723, 32,612,027, and 36,452,156 clean reads were obtained, respectively. After assembly, 70,812, 59,464, and 64,559 unigenes were finally yielded for *A. stoliczkanus*, *T. melanopogon*, and *R. leschenaultii*, respectively. The N_50_ were 1,123, 2,435, and 2,267 bp and the average lengths were 951, 1,080, and 985 bp, respectively.

In addition, a total of 30,596, 26,774, and 27,741 unigenes of *A. stoliczkanus*, *T. melanopogon*, and *R. leschenaultii* were successfully annotated, respectively. After extracting and aligning the putative CDSs, 23,288, 21,680, and 21,050 unigenes with full-length and partial CDSs were annotated for *A. stoliczkanus*, *T. melanopogon*, and *R. leschenaultii*, respectively.

**Fig. 2.**
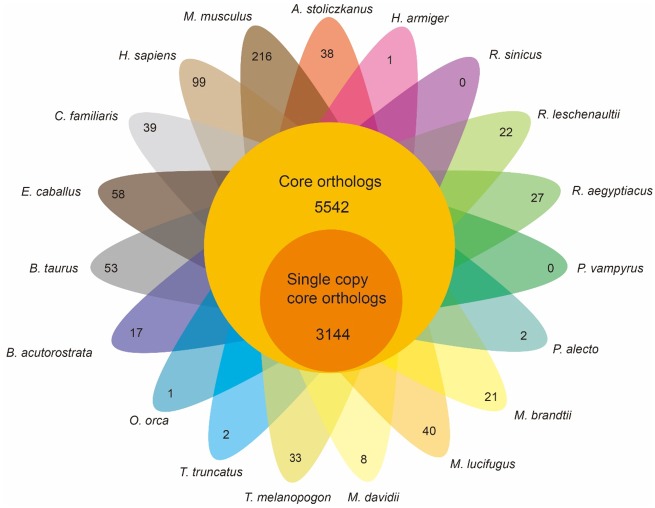
—Venn diagram of orthologous gene analysis for 19 mammals.

A total of 366,267 proteins were obtained from the pooled protein database consisting of the data from the three bat species subjected to RNA-Seq and 16 other mammalian genomes, and then those proteins were binned into 20,637 orthologous groups (gene families). Among these groups, 5,542 gene families were conserved and 3,144 single-copy genes (only one ortholog in each gene family) were identified among these 19 species (fig. 2). After filtering out these 3,144 genes, we eventually obtained 2,833 single-copy orthologous genes.

### Detection of High-Frequency Hearing-Related PSGs

Significant evidence of positive selection was detected along each tested foreground branch. A total of 144 genes were identified to have been under positive selection (supplementary table S2, Supplementary Material online). Separately, 20 genes (32 sites), 23 genes (30 sites), 34 genes (122 sites), 13 genes (37 sites), 24 genes (38 sites), 19 genes (59 sites), and 11 genes (39 sites) were successively identified along seven separate foreground branches, namely, the CF bat branch; FM bat branch; click bat branch; toothed whale branch; CF and FM bat combined branches; CF, FM, and click bat combined branches; and CF, FM, click bat, and toothed whale combined branches, respectively (supplementary table S2, Supplementary Material online). After confirmation of the gene functions, we identified 34 (90 sites) out of 144 PSGs that were closely related to hearing ability or participate in auditory perception. Separately, 9 genes (14 sites) for the CF bat; 7 genes (8 sites) for the FM bat; 6 genes (30 sites) for the click bat; 2 genes (11 sites) for the toothed whale; 5 genes (10 sites) for the CF and FM bat; 2 genes (10 sites) for the CF, FM, and click bat; and 3 genes (7 sites) for the CF, FM, click bat, and toothed whale were identified. Detailed information on PSGs along each separate foreground branch is presented in figure 1 and supplementary table S2, Supplementary Material online.

In detail, the 34 PSGs could be roughly divided into several groups according to their functional information. Among these, 12 PSGs (*CSF1*, *TGFB2*, *TNFRSF11B*, *TNFRSF1A*, *TNFRSF11*, *PIK3R5*, *TYROBP*, *FAM20C*, *UCMA*, *CAT*, *SMPD3*, and *MATN1*) were identified to be related to cochlear development or bony remodeling (Karsenty and Wagner 2002; Teitelbaum and Ross 2003; Aubin et al. 2005; Hyde et al. 2007; Kao et al. 2013; Jenner et al. 2014). Ten PSGs (*SLC4A11*, *CHST3*, *AQP3*, *BBS10*, *ANXA1*, *BCAP31*, *CSF1*, *CALB1*, *TLR4*, and *TNFSF11*) were determined to participate in regulating the concentration of various ions and ion transport activities in the inner ear (Kitahara et al. 2003; Nobentrauth et al. 2003; Desir et al. 2007; Kalinec et al. 2009; Rosenberg et al. 2016). Two PSGs (*GPCR* and *ADGRG1*) are members of the G protein-coupled receptor family responsible for signal transduction (Marinissen and Gutkind 2001; Alexander et al. 2011). In addition, ten PSGs (*TNFRSF11B*, *TNFRSF1A*, *TNFSF11*, *TYROBP*, *TGFB2*, *CSF1*, *CAT*, *PIK3R*, *SDC1*, and *TECPR2*) were also found to be associated with signal transduction (Li and Roberts 2001; Karsenty and Wagner 2002; Teitelbaum and Ross 2003; Kanzaki et al. 2006; Yang et al. 2016). Four PSGs (*GPX2*, *CAT*, *MGST1*, and *TMTC1*) were found to be related to antioxidant activity (Karsenty and Wagner 2002; Kawamoto et al. 2004; Girzalsky et al. 2010; Yang et al. 2016). Moreover, three PSGs (*CEP250*, *BCAP31*, and *SMPD3*) were identified to be related to hearing loss or hearing damage (Khateb et al. 2014; van de Kamp et al. 2015; Rosenberg et al. 2016). Besides, *EMILIN1* was revealed here to have been under positive selection. Previous studies demonstrated that *EMILIN2*, a member of the same gene family, plays a structural role in the cochlear basilar membrane, suggesting that *EMILIN1* may also have an important role in mammalian cochleae (Amma 2003; Selvakumar et al. 2012).

### Parallel/Convergent Evolution Analysis

Large numbers of parallel/convergent genes and sites were identified between all pairs of echolocating mammals. Separately, 154 genes (171 sites), 207 genes (248 sites), 177 genes (203 sites), 119 genes (129 sites), 94 genes (98 sites), and 96 genes (104 sites) were detected to have parallel signals in the comparisons of CF versus FM, CF versus click bat, FM versus click bat, CF versus toothed whale, FM versus toothed whale, and click bat versus toothed whale, respectively. For the convergent signals, 3 genes (3 sites), 6 genes (6 sites), 11 genes (11 sites), 2 genes (2 sites), and 11 genes (11 sites) were detected in the comparisons of CF versus click bat, FM versus click bat, CF versus toothed whale, FM versus toothed whale, and click bat versus toothed whale, respectively. We also detected 45 core genes with parallel sites among all four echolocating mammal lineages (CF, FM, click bat, and toothed whale), although the parallel sites varied among the different comparisons (supplementary table S3, Supplementary Material online). However, no core convergent genes were found among the four types of echolocating mammal.

At the same time, a large number of parallel/convergent genes and sites were detected in pairwise comparisons of echolocating versus nonecholocating species (table 1). In detail, results of paired-sample *t*-test showed that the number of parallel genes and sites detected in comparisons of echolocating species was significantly larger than that identified in their equally phylogenetically distant controls (*P *=* *0.01 and 0.02, respectively). The number of convergent genes and sites detected among echolocating species was always significantly greater than that detected in their equally distant controls (*P *=* *0.04), except for the following two sets of comparisons: (CF vs. FM) versus (CF vs. nonecholocating bat) and (FM vs. toothed whale) versus (FM vs. nonecholocating whale).

### Spatial Distribution of the Positively Selected Sites and Convergent/Parallel Sites in the Protein Structures

The functional domains of each hearing-related PSG were further examined to determine the functional significance of the positively selected sites (supplementary figs. figs. S2 and S3, Supplementary Material online). At the same time, we focused on PSGs with nonrandom parallel/convergent amino acid substitutions; this revealed 17 hearing-related PSGs with parallel sites (supplementary table S4, Supplementary Material online). Structural analysis showed that most of the positively selected sites or the parallel sites were localized in or near the functional regions in the protein structures of those genes (supplementary fig. S2, Supplementary Material online). In three genes, one parallel site of *SLC4A11*, two positively selected sites of *GPCR*, and one positively selected site of *TMTC1* were located within the protein transmembrane domain of the corresponding genes. In addition, most of the positively selected sites and parallel sites were localized in the functional or repeats domains of *TNFRSF1A*, *TNFRSF11B*, *FAM20C*, *GSF1*, *TECPR2*, *CAT*, *ADGRG1*, *TGFB2*, *ANXA1*, *GPCR*, *MATN1*, and *TMTC1*. Furthermore, for *TNFRSF1A*, one positively selected site (84) was at an antiparallel homodimerization interface and a site for polypeptide ligand binding, one positively selected site (143) was at a parallel homodimerization interface, and one parallel site (73) was at an antiparallel homodimerization interface. The positively selected sites 177 and 298 of the *CAT* gene were located at the NADPH binding site and heme binding pocket, respectively. For the *TGFB2* gene, the positively selected site 295 (detected along the click bat branch) was also identified as the parallel site (in the CF vs. click bat comparison).

**Fig. 3.**
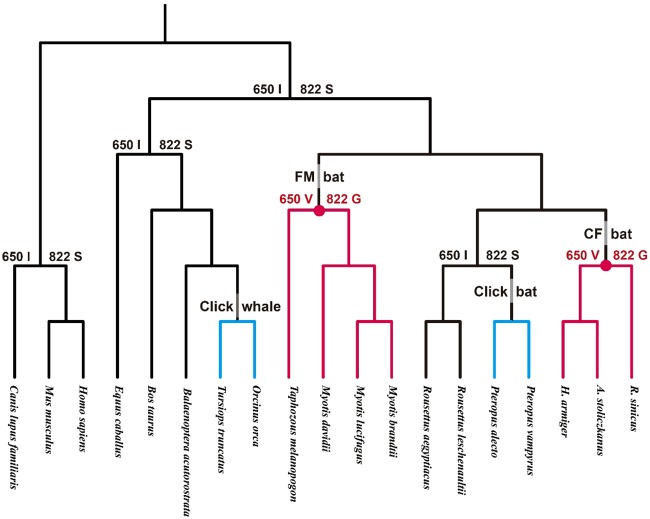
—Evolutionary trajectory of parallel sites from TECPR2 mapped onto the species phylogeny. Red branches indicate laryngeal echolocating bats and blue ones indicate click echolocating bats and whales. Red points indicate the nodes where higher frequency hearing might have been acquired.

Notably, CF and FM bats with higher frequency hearing shared the same amino acid at two sites, 650 and 822, on the TECPR2 protein sequence. These two sites were under positively selected along the CF and FM combined foreground branches and they were also detected to be the parallel-evolved sites in the CF versus FM comparison, indicating that these two sites are functionally important and have adaptively evolved in CF and FM bats. To obtain insights into the evolutionary trajectory of these two sites for the *TECPR2* gene, we reconstructed the ancestral amino acids of the two sites and mapped them onto the species’ phylogenetic tree (fig. 3). This indicated that the two sites have the same amino acid substitutions in all laryngeal echolocating bats with high-frequency hearing.

### Key Hearing-Related Physiological Terms and Pathways

Several hearing-related GO terms were significantly enriched by the 34 PSGs, including terms related to peroxidase and oxidoreductase activity (GO: 0004601 and GO: 0016684), antioxidant activity (GO: 0016209), homeostatic process (GO: 0042592), multicellular organismal homeostasis (GO: 0048871), tissue homeostasis (GO: 0001894), anatomical structure homeostasis (GO: 0060249), and ossification (GO: 0001503) (supplementary table S5, Supplementary Material online). Three other GO terms were significantly associated with seven PSGs along the FM branch, including organ morphogenesis (GO: 0009887), anatomical structural morphogenesis (GO: 0009653), and multicellular organismal homeostasis (GO: 0048871).

**Fig. 4.**
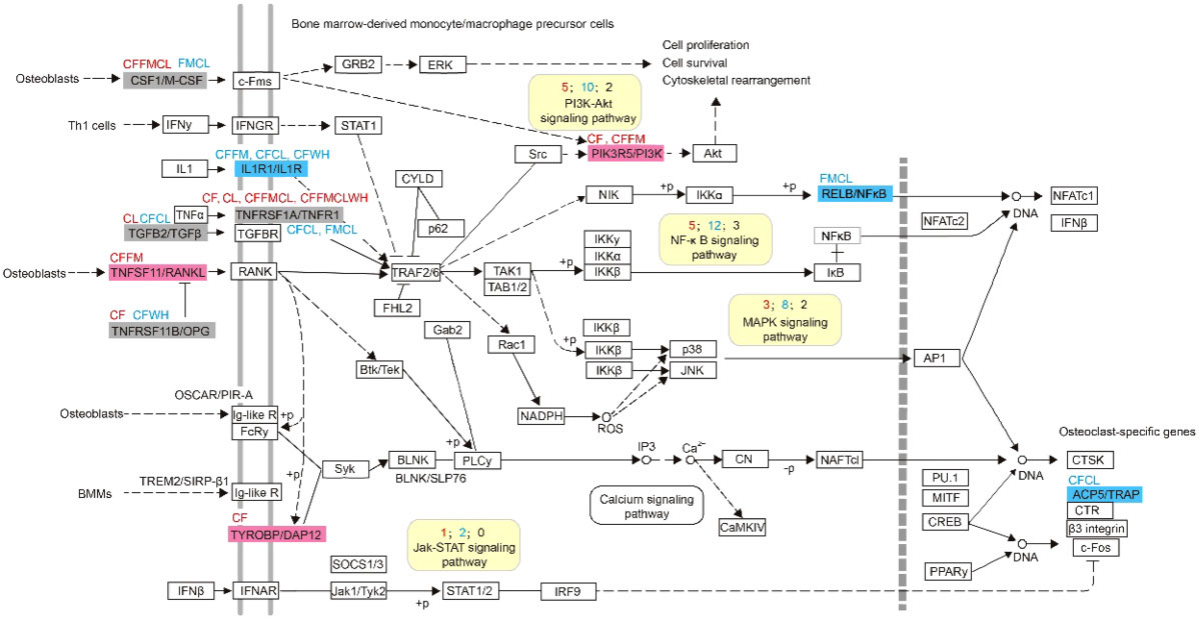
—Positively selected genes and parallel genes involved in the osteoclast differentiation pathway (ko04380). Genes with positively selected sites, parallel sites, and both are shown with pink, blue, and gray rectangles, respectively. Foreground branches tested for PSGs and pairwise compared branches tested for parallel genes are shown above and below the rectangles with red and blue typeface, respectively. Yellow rectangles indicate signaling pathways involved in this pathway, the number of PSGs, parallel genes, and genes with both positively selected sites and parallel sites are shown in red, blue, and black, respectively. For convenience, CF, FM, CL, and WH standing for CF bats, FM bats, click bats, and echolocating toothed whales, respectively, are shown in this figure.

In addition, one KEGG pathway related to cochlear development, namely, osteoclast differentiation (ko04380), was significantly enriched by different groups of PSGs (supplementary table S5, Supplementary Material online). We then mapped the PSGs and parallel genes into this pathway and found that seven PSGs and seven parallel genes participated in it (fig. 4). Four other signaling-related pathways were also found to interact with the osteoclast differentiation pathway (ko04380), namely, the NF-κB signaling pathway (ko04064), MAPK signaling pathway (ko04010), PI3K–Akt signaling pathway (ko04151), and Jak–STAT signaling pathway (ko04630). Several PSGs and parallel genes were also detected in these four signaling pathways. To obtain insights into the relationships of PSGs and parallel genes in the osteoclast differentiation (ko04380) pathway, a protein interaction network was created, as shown in supplementary figure S4, Supplementary Material online. Besides, one signaling molecule and interaction pathway, namely, cytokine–cytokine receptor interaction (ko04060), and four signal transduction-related pathways, namely, sphingolipid signaling pathway (ko04071), FoxO signaling pathway (ko04068), NF-κB signaling pathway (ko04064), and MAPK signaling pathway (ko04010), were also found to be significantly enriched by different groups of PSGs (supplementary table S5, Supplementary Material online).

**Fig. 5.**
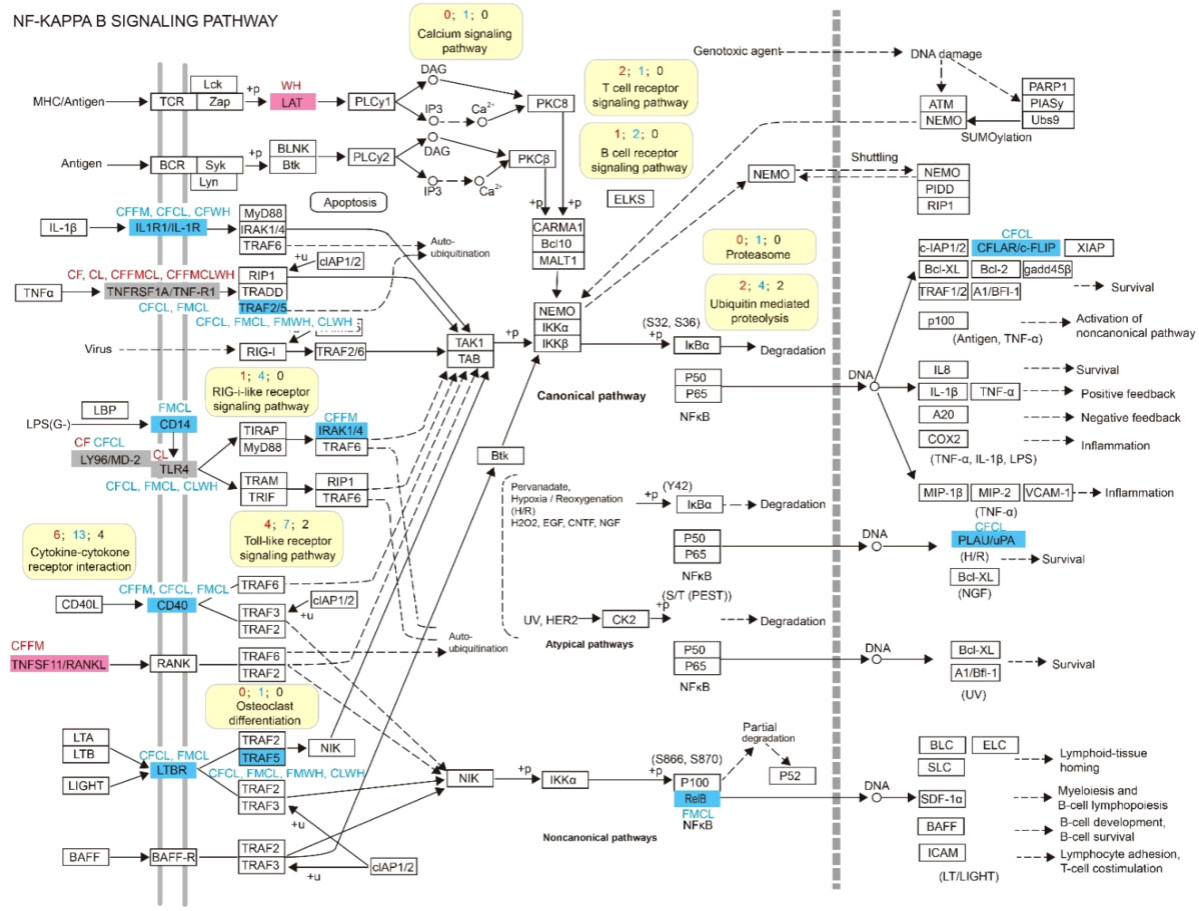
—Positively selected genes and parallel genes involved in the NF-κB signaling pathway (ko04064). Other information in this figure is similar to that in figure 4.

With regard to the different groups of parallel/convergent genes, 45 core parallel genes among the four echolocating lineages were significantly enriched in the NF-κB signaling pathway (ko04064), indicating the functional importance of this pathway for all tested echolocating mammals. Given that this pathway also closely interacts with osteoclast differentiation (ko04380), we then carefully analyzed this pathway and found that 5 PSGs and 12 parallel genes are involved in it (fig. 5). A protein interaction network corresponding to these was created (supplementary fig. S5, Supplementary Material online) to visualize the relationships of these genes.

## Discussion

Comparative genomic analyses have been widely used to unveil the genetic bases underlying specific adaptations of organisms and phenotypic traits (Yim et al. 2014; Kober and Pogson 2017; Gayk et al. 2018). Transcriptomic sequencing is an effective approach to detect genes that are abundantly expressed in specific tissues (Mitterboeck et al. 2017; Tong et al. 2017). In this study, we combined these two methods, sequenced and assembled the cochlear transcriptomes of three different types of echolocating bats, and then combined them with the genomic data of another 16 mammals with or without the ability to hear high-frequency sounds. Our analyses provide strong evidence that several hearing-related genes have undergone adaptive evolution in four different lineages of echolocating animals. Furthermore, as described below, this study revealed specific physiological adaptations in the cochleae of echolocators.

### Adaptive Evolution of Cochlear Bony Development-Related Genes

Normal hearing requires exquisite cooperation between bony and sensorineural structures within the cochlea (Akil 2014). Cochlear structure and bone density are essential for the transduction of acoustic signals and perception and are influenced by the balance between bone resorption by osteoclasts and formation by osteoblasts (Teitelbaum 2000; Horner 2012). Our analyses provide strong evidence that 12 genes involved in cochlear bony development have been subjected to adaptive evolution in high-frequency hearing mammals: *TNFRSF11B*, *TNFRSF1A*, *TNFSF11*, *PIK3R5*, *FAM20C*, *TYROBP*, *TGFB2*, *UCMA*, *CSF1*, *CAT*, *SMPD3*, and *MATN1*. In the different pairwise comparisons among echolocators, most of these PSGs were revealed to have signals of parallel evolution. Furthermore, functional enrichment analyses showed that seven of these genes (*TNFRSF11B*, *TNFRSF1A*, *TNFSF11*, *PIK3R5*, *CSF1*, *TYROBP*, and *TGFB2*) are significantly enriched in the osteoclast differentiation (ko04380) pathway (fig. 4) and four of them (*FAM20C*, *UCMA*, *CSF1*, and *CAT*) are significantly enriched in ossification (GO: 0001503). Both ossification and osteoclast differentiation are related to cochlear bony development, indicating that this dynamic process has undergone adaptive change in mammals that can hear high-frequency sounds.

Moreover, the adaptive changes in cochlear bony development appear to have been more intensive in echolocating bats than those in echolocating toothed whales, as all of the associated PSGs were detectable when echolocating bats were included in the foreground branches, but only two (*TNFRSF1A* and *UCMA*) of them were detected when echolocating toothed whales were included in the foreground branches.

Besides PSGs and parallel evolved genes, four signaling pathways involved in osteoclast differentiation (ko04380) were also detected. Because NF-κB is one of the osteoclastogenic transcription factors, and the NF-κB signaling pathway (ko04064) was found to be significantly enriched by multiple gene sets (supplementary table S5, Supplementary Material online), especially for 45 parallel genes (with parallel sites along all of the tested echolocating lineages), it is indicated that this signaling pathway is functionally the most important for all echolocating bats and whales.

### Antioxidant Activity and Hearing Protection

Acoustic overstimulation is traumatic for cochlear cells and compromises auditory function (Hu et al. 2000; Zine and van de Water 2004; Yang et al. 2016). Chronic exposure to high-intensity noise leads to hearing loss, the damage of which usually begins in the high-frequency regions (Tarter and Robins 1990; Yang et al. 2016). A potential mechanism behind the hearing loss due to acoustic overstimulation is the generation of reactive oxygen species (ROS) (Sha et al. 2001). ROS that are not removed by antioxidant defenses would be expected to cause significant damage to the sensory cells of the cochlea (Clerici et al. 1996; Yamasoba et al. 1998). Outer hair cells, specifically those at the base of the cochlea that usually respond to high-frequency signals, appear to be highly sensitive to damage due to ROS compared with supporting cells (Sha et al. 2001; Yang et al. 2015). For the survival of echolocating bats and whales, it is important to maintain the sensitivity of high-frequency hearing. Therefore, we suppose that effective antioxidant activity in the cochlea of echolocating mammals is more crucial than that in nonecholocating mammals; as such, antioxidant-related genes might have been subjected to particular natural selection in echolocators. In line with this, in this study three antioxidant-related genes (*GPX2*, *CAT*, and *MGST1*), which were significantly enriched in peroxidase activity (GO: 0004601), oxidoreductase activity, acting on peroxide as an acceptor (GO: 0016684), and antioxidant activity (GO: 0016209), were found to have been under positive selection. Moreover, *TMTC1*, another positively selected gene, was among proteins containing TPRs which were involved in a variety of biological processes including peroxisomal protein transport, and was also found to be potentially related to antioxidant activity (Girzalsky et al. 2010).

Moreover, these four genes (*GPX2*, *CAT*, *MGST1*, and *TMTC1*) were found to have been under positive selection when the echolocating bats were included in the foreground branches, suggesting that antioxidant-related genes may have been subjected to stronger selective pressures in echolocating bats than in echolocating toothed whales. This could be explained by the fact that echolocating bats, especially laryngeal echolocating bats, are heavily dependent on acoustic signals in daily life, for which the ability to perceive high-frequency signals is essential. This could in turn be jeopardized by ROS, so their antioxidant capacity is a key characteristic.

Several studies have also found that in the inner ear, the NF-κB signaling pathway (ko04064) can rapidly respond to ototoxic stimulants, such as noise and ototoxic drugs, to protect hair cells and spiral ganglion cells (Jiang et al. 2005; Nagashima et al. 2007). The MAPK signaling pathway (ko04010) has also been shown to protect the inner ear against stress induced by noise and ototoxicity (Bell and Oberholtzer 2010; Jamesdaniel et al. 2011). In this study, we found several positively selected and parallel evolved genes that were individually significantly enriched in these two pathways, suggesting that adaptive changes of physiological processes have taken place to defend against potential oxidation damage in the cochlea of echolocating mammals. Three other genes (*CEP250*, *SMPD3*, and *BCAP31*) that were found to be related to hearing damage with or without parallel sites were also found to have been under positive selection, suggesting their potential adaptive changes and effects on echolocating bats and whales.

### Ion Transport and Cochlear Homeostasis

The transduction of sound into nerve impulses requires an ionic environment that depends on a variety of ion transport processes in epithelial and endothelial cells of the cochlea (Wangemann and Marcus 2017). Electrochemical gradients and homeostatic processes are important for various ion transport activities (Nin et al. 2016; Wangemann and Marcus 2017). Ten PSGs were found to be related to ion transport and homeostasis. In the inner ear, *SLC4A11* was shown to be involved in the transport of potassium through the fibrocyte layer to the stria vascularis and essential for the generation of endocochlear potential (Desir et al. 2007; Gröger et al. 2010). It is also involved in borate homeostasis. The other nine PSGs (*CHST3*, *AQP3*, *BBS10*, *ANXA1*, *BCAP31*, *CSF1*, *CALB1*, *TLR4*, and *TNFSF11*) were found to be significantly enriched in terms related to homeostasis, including homeostatic process, multicellular organismal homeostasis, tissue homeostasis, and anatomical structure homeostasis (supplementary table S5, Supplementary Material online). Therefore, our results reveal the unique physiological and molecular mechanisms underlying homeostasis in the cochlea of echolocating bats and whales.

### Adaptive Change of Signal Transduction Genes and Pathways

The transduction of numerous signals involved in various physiological processes is an important basis for auditory function. Adaptive changes in specific genes and specific signaling pathways may influence several corresponding physiological processes. For example, in this study, the NF-κB signaling pathway (ko04064) was found to be involved in the process of osteoclast differentiation and also affected antioxidant activity. Moreover, the PSGs that were significantly enriched in the cytokine–cytokine receptor interaction pathway (ko04060) were all found to be involved in the osteoclast differentiation pathway (ko04380) (supplementary table S5, Supplementary Material online). Moreover, signaling transduction-related PSGs that were found to be significantly enriched in the sphingolipid signaling pathway (ko04071), MAPK signaling pathway (ko04010), and TNF signaling pathway (ko04668) were all found in the osteoclast differentiation pathway (ko04380) (supplementary table S5, Supplementary Material online). All of the above findings suggested that the adaptive evolution of genes in corresponding signaling pathways may have positive effects on multiple crucial hearing-related physiological processes in echolocating bats and whales. In other words, most of the adaptive evolution took place in the signaling regulation-related genes, which can also participate in specific hearing-related physiological processes.

### Convergent/Parallel Evolution in Bats and Whales

A range of studies of individual genes have revealed that molecular convergence is more common among echolocating mammals than previously thought (Li et al. 2010; Davies et al. 2012; Shen et al. 2012; Thomas and Hahn 2015). However, no genome-wide protein sequence convergence among echolocators was proven by the following studies. Researchers found that the genome-wide phylogenetic signal of molecular convergence is not stronger for echolocators than that for comparable nonecholocating species (Thomas and Hahn 2015; Zou and Zhang 2015). Our study showed that no hearing-related functional categories were significantly enriched by those parallel/convergent genes, although the parallel/convergent signals among echolocating species were always stronger than those from their equally distant controls. While in other study, Liu et al. (2018) found that the number of convergent genes between echolocating bats and toothed whales is not significantly different from that between their comparable nonecholocating lineages; however, there is significant convergence in hearing-genes between echolocating bats and toothed whales. It thus remains an open question whether molecular convergence that underlies the echolocating phenotype is widespread among echolocators. Further analysis on numerous species from various echolocating and nonecholocating taxa is needed and further evidence of molecular convergence should be carefully examined with a focus on specific hearing-related genes and associated molecular changes.

In addition, although a number of convergent/parallel genes and sites were detected between all pairs of echolocators, no convergent/parallel sites were found to have undergone positive selection, with the exceptions of 295 in TGFB2 and 650 and 822 in TECPR2 (supplementary fig. S2, Supplementary Material online). Moreover, no specific hearing-related physiological terms were found to be significantly enriched by separate convergent/parallel gene sets from the six echolocating pairwise comparisons. All of these results suggest that these convergent/parallel signals may have been caused by neutral evolution (Zou and Zhang 2015).

Therefore, to characterize the molecular basis of echolocation, comparative methods should be used, such as integrated analyses of convergent/parallel and positive selection (Hu et al. 2017). In addition, downstream analysis of the corresponding gene set is also necessary. Fortunately, we found a set of 45 genes with parallel sites among all types of echolocator that were significantly enriched in the NF-κB signaling pathway (ko04064), which is also significantly enriched by hearing-related PSGs. This pathway is involved in osteoclast differentiation, antioxidant activity, and protection of the cochlea, indicating that these physiological processes have undergone adaptive change in echolocating bats and whales.

Moreover, changes in the structure of cochlea during development may also be under convergent/parallel and adaptive evolution in echolocators because genes expressed in the cochlea might differ during different developmental stages of echolocators (Mao et al. 2017). In our study, we only obtained transcriptomes from adult bats, so were unable to evaluate the levels of convergent/parallel evolution of those cochlear genes only expressed in other developmental stages of echolocators. Therefore, in further study, there is a need to analyze the transcriptomes of echolocators at different developmental stages.

### Important Candidate Gene for Laryngeal Echolocating Bats

Most of the parallel sites detected in PSGs differed from the sites under positive selection as revealed by our results. Convergent/parallel sites were defined as those that had undergone adaptive convergent/parallel evolution if they were the same as those sites that had undergone positive selection. Although widely convergent/parallel sites were detected, adaptive parallel signals were rare. Intriguingly, for the *TECPR2* gene, two parallel sites, 650 and 822, detected in CF and FM bats were found to be under positive selection (when combined CF and FM bats as a foreground branch), indicating that these two parallel sites were fixed by natural selection. However, there is limited knowledge about *TECPR2*, except that it is a signaling transduction-related gene that is expressed in the cochlea (Li and Roberts 2001). Detailed information about the relationship between *TECPR2* and the hearing process is still limited, but our study indicated its functional importance in laryngeal echolocating bats. Figure 3 shows that all laryngeal echolocating bats with higher frequency hearing share the same amino acid at these two sites, 650 V and 822 G. Furthermore, the fact that the 650 site is located in a region of beta-propeller repeats suggested that this amino acid replacement may introduce subtle changes in the functional activity of TECPR2 proteins and consequently contribute to changes in signal transduction in CF and FM bats. Previous studies demonstrated the involvement of the replacement of similar adaptive parallel amino acids in two key genes (*Prestin* and *KCNQ4*) in high-frequency hearing (Liu et al. 2011). Researchers found that the evolutionary trajectories of the parallel sites in *Prestin* and *KCNQ4* suggest the independent acquisition of the ability to hear higher frequency sounds in laryngeal echolocating bats, which may also apply to *TECPR2*. The occurrence of parallel evolved sites in these genes also suggests that higher frequency hearing probably developed further after its origin in bats.

## Conclusion

High-frequency hearing confers a survival benefit to many animals and is essential for the daily life of echolocating bats and whales. Among the genes expressed in the cochlea, 144 genes were found to be under positive selection in different types of echolocating bats and whales. Among them, 34 out of 144 PSGs were identified to be related to hearing behavior or auditory processes. The other 110 PSGs may also be important for high-frequency hearing, but there is currently a lack of empirical evidence for this, thus more advanced research is needed. Further analyses of 34 hearing-related PSGs indicated that multiple features have adaptively evolved in echolocating bats and whales, including cochlear bony development, antioxidant activity, ion balance, and homeostatic processes, as well as signal transduction. Notably, we also found two adaptive parallel evolved sites in *TECPR2*, which could be important for the evolution of echolocation and ultrasonic hearing in laryngeal echolocating bats. Our study provides for the first time a comprehensive understanding of the genetic basis underlying high-frequency hearing in the cochlea of echolocating bats and whales. This study also reveals a large number of candidate genes responsible for echolocation and ultrasonic hearing, which warrant further study. 

## Supplementary Material

Supplementary data are available at *Genome Biology and Evolution* online.

## Supplementary Material

evz250_Supplementary_Data

## Notes

**Data deposition:** Raw sequence data have already been deposited into the NCBI Sequence Read Archive database (SRA run accession numbers: *A. stoliczkanus*, SRS3011421; *T. melanopogon*, SRS3011514; *R. leschenaultii*, SRS3011407), and these data have been made available now.

## References

[evz250-B2] Akil O. 2014 Disrupted bone remodeling leads to cochlear overgrowth and hearing loss in a mouse model of fibrous dysplasia. PLoS One 9(5):e94989. 24788917 10.1371/journal.pone.0094989 PMC4006800

[evz250-B3] Alexander S, Mathie A, Peters JA. 2011 G protein‐coupled receptors. Brit J Pharmacol. 164:S5–S113.

[evz250-B4] Amma LL. 2003 An EMILIN family extracellular matrix protein identified in the cochlear basilar membrane. Mol Cell Neurosci. 23(3):460–472. 12837629 10.1016/s1044-7431(03)00075-7

[evz250-B5] Au WW, Simmons JA. 2007 Echolocation in dolphins and bats. Phys Today. 60(9):40–45.

[evz250-B6] Aubin I, et al 2005 A deletion in the gene encoding sphingomyelin phosphodiesterase 3 (Smpd3) results in osteogenesis and dentinogenesis imperfecta in the mouse. Nat Genet. 37(8):803–805. 16025116 10.1038/ng1603

[evz250-B7] Bell TJ, Oberholtzer JC. 2010 cAMP-induced auditory supporting cell proliferation is mediated by ERK MAPK signaling pathway. J Assoc Res Otolaryngol. 11(2):173–185. 20107853 10.1007/s10162-009-0205-8 PMC2862916

[evz250-B10] Cantarel BL, et al 2007 MAKER: an easy-to-use annotation pipeline designed for emerging model organism genomes. Genome Res. 18(1):188–196. 18025269 10.1101/gr.6743907 PMC2134774

[evz250-B11] Castresana J. 2000 Selection of conserved blocks from multiple alignments for their use in phylogenetic analysis. Mol Biol Evol. 17(4):540–552. 10742046 10.1093/oxfordjournals.molbev.a026334

[evz250-B12] Chen MY, Liang D, Zhang P. 2017 Phylogenomic resolution of the phylogeny of Laurasiatherian mammals: exploring phylogenetic signals within coding and noncoding sequences. Genome Biol Evol. 9(8):1998–2012. 28830116 10.1093/gbe/evx147 PMC5737624

[evz250-B14] Churchill M, Martinez-Caceres M, de Muizon C, Mnieckowski J, Geisler JH. 2016 The origin of high-frequency hearing in whales. Curr Biol. 26(16):2144–2149. 27498568 10.1016/j.cub.2016.06.004

[evz250-B15] Clerici WJ, Hensley K, Dimartino DL, Butterfield DA. 1996 Direct detection of ototoxicant-induced reactive oxygen species generation in cochlear explants. Hearing Res. 98(1–2):116–124. 10.1016/0378-5955(96)00075-5 8880186

[evz250-B16] Conesa A, et al 2005 Blast2GO: a universal tool for annotation, visualization and analysis in functional genomics research. Bioinformatics 21(18):3674–3676. 16081474 10.1093/bioinformatics/bti610

[evz250-B17] Dallos P, Fakler B. 2002 PRESTIN, a new type of motor protein. Nat Rev Mol Cell Biol. 3(2):104–111. 11836512 10.1038/nrm730

[evz250-B18] Davies K, Cotton J, Kirwan J, Teeling E, Rossiter S. 2012 Parallel signatures of sequence evolution among hearing genes in echolocating mammals: an emerging model of genetic convergence. Heredity 108(5):480–489. 22167055 10.1038/hdy.2011.119 PMC3330687

[evz250-B19] Dawkins R editor. 1986 The blind watchmaker. Essex (United Kingdom): Longman Scientific and Technical.

[evz250-B21] Desir J, et al 2007 Borate transporter SLC4A11 mutations cause both Harboyan syndrome and non-syndromic corneal endothelial dystrophy. J Med Genet. 44(5):322–326. 17220209 10.1136/jmg.2006.046904 PMC2597979

[evz250-B22] Foote AD, et al 2015 Convergent evolution of the genomes of marine mammals. Nat Genet. 47(3):272–275. 25621460 10.1038/ng.3198 PMC4644735

[evz250-B24] Gayk ZG, Le DD, Horn J, Lindsay AR. 2018 Genomic insights into natural selection in the common loon (*Gavia immer*): evidence for aquatic adaptation. BMC Evol Biol. 18(1):64. 29703132 10.1186/s12862-018-1181-6 PMC5921391

[evz250-B25] Girzalsky W, Saffian D, Erdmann R. 2010 Peroxisomal protein translocation. Biochim Biophys Acta. 1803:724–731. 20079383 10.1016/j.bbamcr.2010.01.002

[evz250-B26] Grabherr MG, et al 2011 Full-length transcriptome assembly from RNA-Seq data without a reference genome. Nat Biotechnol. 29(7):644–652. 21572440 10.1038/nbt.1883 PMC3571712

[evz250-B28] Gröger N, et al 2010 SLC4A11 prevents osmotic imbalance leading to corneal endothelial dystrophy, deafness, and polyuria. J Biol Chem. 285(19):14467–14474. 20185830 10.1074/jbc.M109.094680 PMC2863209

[evz250-B29] Horner KC. 2012 Bone and the ear In: Bronner F, Farach-Carson MC, Roach HI, editors. Bone-metabolic functions and modulators. New York: Springer p. 251–269.

[evz250-B30] Hu BH, Guo W, Wang PY, Henderson D, Jiang SC. 2000 Intense Noise-induced apoptosis in air cells of guinea pig cochleae. Acta Oto-Laryngol. 120(1):19–24. 10779180

[evz250-B31] Hu Y, et al 2017 Comparative genomics reveals convergent evolution between the bamboo-eating giant and red pandas. Proc Natl Acad Sci U S A. 114(5):1081–1086. 28096377 10.1073/pnas.1613870114 PMC5293045

[evz250-B32] Hyde G, Dover S, Aszodi A, Wallis GA, Boot-Handford RP. 2007 Lineage tracing using matrilin-1 gene expression reveals that articular chondrocytes exist as the joint interzone forms. Dev Biol. 304(2):825–833. 17313942 10.1016/j.ydbio.2007.01.026 PMC2795868

[evz250-B33] Iseli C, Jongeneel CV, Bucher P. 1999 ESTScan: a program for detecting, evaluating, and reconstructing potential coding regions in EST sequences. Proc Int Conf Intell Syst Mol Biol. 99:138–148. 10786296

[evz250-B34] Jamesdaniel S, et al 2011 Noise induced changes in the expression of p38/MAPK signaling proteins in the sensory epithelium of the inner ear. J Proteomics. 75(2):410–424. 21871588 10.1016/j.jprot.2011.08.007 PMC3225708

[evz250-B35] Jenner F, et al 2014 Differential gene expression of the intermediate and outer interzone layers of developing articular cartilage in murine embryos. Stem Cells Dev. 23(16):1883–1898. 24738827 10.1089/scd.2013.0235 PMC4120811

[evz250-B36] Jiang H, Sha SH, Schacht J. 2005 NF-kappaB pathway protects cochlear hair cells from aminoglycoside-induced ototoxicity. J Neurosci Res. 79(5):644–651. 15672440 10.1002/jnr.20392

[evz250-B37] Jones G, Teeling EC. 2006 The evolution of echolocation in bats. Trends Ecol Evol. 21(3):149–156. 16701491 10.1016/j.tree.2006.01.001

[evz250-B38] Kalinec F, et al 2009 Glucocorticoid-stimulated, transcription-independent release of annexin A1 by cochlear Hensen cells. Br J Pharmacol. 158(7):1820–1834. 19912231 10.1111/j.1476-5381.2009.00473.x PMC2801223

[evz250-B39] Kanzaki S, Ito M, Takada Y, Ogawa K, Matsuo K. 2006 Resorption of auditory ossicles and hearing loss in mice lacking osteoprotegerin. Bone 39(2):414–419. 16564235 10.1016/j.bone.2006.01.155

[evz250-B40] Kao SY, et al 2013 Loss of osteoprotegerin expression in the inner ear causes degeneration of the cochlear nerve and sensorineural hearing loss. Neurobiol Dis. 56:25–33. 23607938 10.1016/j.nbd.2013.04.008 PMC3752395

[evz250-B41] Karsenty G, Wagner EF. 2002 Reaching a genetic and molecular understanding of skeletal development. Dev Cell. 2(4):389–406. 11970890 10.1016/s1534-5807(02)00157-0

[evz250-B42] Kawamoto K, et al 2004 Antioxidant gene therapy can protect hearing and hair cells from ototoxicity. Mol Ther. 9(2):173–181. 14759801 10.1016/j.ymthe.2003.11.020

[evz250-B43] Khateb S, et al 2014 A homozygous nonsense CEP250 mutation combined with a heterozygous nonsense C2orf71 mutation is associated with atypical Usher syndrome. J Med Genet. 51(7):460–469. 24780881 10.1136/jmedgenet-2014-102287

[evz250-B44] Kitahara T, Fukushima M, Uno Y, Mishiro Y, Kubo T. 2003 Up-regulation of cochlear aquaporin-3 mRNA expression after intra-endolymphatic sac application of dexamethasone. Neurol Res. 25(8):865–870. 14669532 10.1179/016164103771953989

[evz250-B45] Kober KM, Pogson GH. 2017 Genome-wide signals of positive selection in strongylocentrotid sea urchins. BMC Genomics 18(1):555. 28732465 10.1186/s12864-017-3944-7 PMC5521101

[evz250-B46] Li D, Roberts R. 2001 WD-repeat proteins: structure characteristics, biological function, and their involvement in human diseases. Cell Mol Life Sci. 58(14):2085–2097. 11814058 10.1007/PL00000838 PMC11337334

[evz250-B47] Li G, et al 2008 The hearing gene *Prestin* reunites echolocating bats. Proc Natl Acad Sci U S A. 105(37):13959–13964. 18776049 10.1073/pnas.0802097105 PMC2544561

[evz250-B48] Li L, Stoeckert CJ, Roos DS. 2003 OrthoMCL: identification of ortholog groups for eukaryotic genomes. Genome Res. 13(9):2178–2189. 12952885 10.1101/gr.1224503 PMC403725

[evz250-B49] Li W, Godzik A. 2006 Cd-hit: a fast program for clustering and comparing large sets of protein or nucleotide sequences. Bioinformatics 22(13):1658–1659. 16731699 10.1093/bioinformatics/btl158

[evz250-B50] Li Y, Liu Z, Shi P, Zhang J. 2010 The hearing gene Prestin unites echolocating bats and whales. Curr Biol. 20(2):R55–R56. 20129037 10.1016/j.cub.2009.11.042 PMC11646320

[evz250-B51] Liu Y, et al 2010 Convergent sequence evolution between echolocating bats and dolphins. Curr Biol. 20(2):R53–R54. 20129036 10.1016/j.cub.2009.11.058

[evz250-B52] Liu Z, et al 2011 Parallel evolution of *KCNQ4* in echolocating bats. PLoS One 6(10):e26618. 22046315 10.1371/journal.pone.0026618 PMC3200345

[evz250-B54] Liu Z, Qi F, Xu D, Zhou X, Shi P. 2018 Genomic and functional evidence reveals molecular insights into the origin of ehcolocation in whales. Sci Adv. 4(10):eaat8821. 30306134 10.1126/sciadv.aat8821 PMC6170035

[evz250-B55] Löytynoja A, Goldman N. 2010 webPRANK: a phylogeny-aware multiple sequence aligner with interactive alignment browser. BMC Bioinformatics 11 (1):7. 21110866 10.1186/1471-2105-11-579 PMC3009689

[evz250-B56] Madsen PT, Kerr I, Payne R. 2004 Echolocation clicks of two free-ranging, oceanic delphinids with different food preferences: false killer whales *Pseudorca crassidens* and Risso’s dolphins *Grampus griseus*. J Exp Biol. 207(11):1811–1823. 15107437 10.1242/jeb.00966

[evz250-B57] Mao B, Moss CF, Wilkinson GS. 2017 Age-dependent gene expression in the inner ear of big brown bats (*Eptesicus fuscus*). PLoS One 12(10):e0186667. 29073148 10.1371/journal.pone.0186667 PMC5658057

[evz250-B58] Marinissen MJ, Gutkind JS. 2001 G-protein-coupled receptors and signaling networks: emerging paradigms. Trends Pharmacol Sci. 22(7):368–376. 11431032 10.1016/s0165-6147(00)01678-3

[evz250-B61] Mcgowen MR. 2011 Toward the resolution of an explosive radiation-a multilocus phylogeny of oceanic dolphins (Delphinidae). Mol Phylogenet Evol. 60(3):345–357. 21600295 10.1016/j.ympev.2011.05.003

[evz250-B62] Mcgowen MR, Spaulding M, Gatesy J. 2009 Divergence date estimation and a comprehensive molecular tree of extant cetaceans. Mol Phylogenet Evol. 53(3):891–906. 19699809 10.1016/j.ympev.2009.08.018

[evz250-B63] Mitterboeck TF, et al 2017 Positive and relaxed selection associated with flight evolution and loss in insect transcriptomes. Gigascience 6(10):1–14. 10.1093/gigascience/gix073 PMC5632299 29020740

[evz250-B64] Nagashima R, et al 2007 Acoustic overstimulation facilitates the expression of glutamate–cysteine ligase catalytic subunit probably through enhanced DNA binding of activator protein-1 and/or NF-κB in the murine cochlea. Neurochem Int. 51(2–4):209–215. 17559975 10.1016/j.neuint.2007.04.023

[evz250-B65] Neuweiler G. 1989 Foraging ecology and audition in echolocating bats. Trends Ecol Evol. 4(6):160–166. 21227342 10.1016/0169-5347(89)90120-1

[evz250-B66] Neuweiler G. 1990 Auditory adaptations for prey capture in echolocating bats. Physiol Rev. 70(3):615–641. 2194220 10.1152/physrev.1990.70.3.615

[evz250-B67] Nin F, et al 2016 The unique electrical properties in an extracellular fluid of the mammalian cochlea; their functional roles, homeostatic processes, and pathological significance. Pflugers Arch Eur J Physiol. 468(10):1637–1649. 27568193 10.1007/s00424-016-1871-0 PMC5026722

[evz250-B68] Nobentrauth K, Zheng QY, Johnson KR. 2003 Association of cadherin 23 with polygenic inheritance and genetic modification of sensorineural hearing loss. Nat Genet. 35:21–23. 10.1038/ng1226 PMC2864026 12910270

[evz250-B69] Rosenberg C, et al 2016 Genomic copy number alterations in non‐syndromic hearing loss. Clin Genet. 89(4):473–477. 26456090 10.1111/cge.12683

[evz250-B70] Salorio-Corbetto M, Baer T, Moore B. 2017 Quality ratings of frequency-compressed speech by participants with extensive high-frequency dead regions in the cochlea. Int J Audiol. 56(2):106–120. 27724057 10.1080/14992027.2016.1234071 PMC5283379

[evz250-B71] Selvakumar D, et al 2012 CNGA3 is expressed in inner ear hair cells and binds to an intracellular C-terminus domain of EMILIN1. Biochem J. 443(2):463–476. 22248097 10.1042/BJ20111255 PMC6363478

[evz250-B72] Sha SH, Taylor R, Forge A, Schacht J. 2001 Differential vulnerability of basal and apical hair cells is based on intrinsic susceptibility to free radicals. Hearing Res. 155(1–2):1–8. 10.1016/s0378-5955(01)00224-6 11335071

[evz250-B73] Shen YY, Liang L, Li GS, Murphy RW, Zhang YP. 2012 Parallel evolution of auditory genes for echolocation in bats and toothed whales. PLoS Genet. 8(6):e1002788. 22761589 10.1371/journal.pgen.1002788 PMC3386236

[evz250-B76] Stockel D, et al 2016 Multi-omics enrichment analysis using the GeneTrail2 web service. Bioinformatics 32:1502–1508. 26787660 10.1093/bioinformatics/btv770

[evz250-B77] Stoffberg S, Jacobs DS, Mackie IJ, Matthee CA. 2010 Molecular phylogenetics and historical biogeography of *Rhinolophus* bats. Mol Phylogenet Evol. 54(1):1–9. 19766726 10.1016/j.ympev.2009.09.021

[evz250-B79] Tarter SK, Robins TG. 1990 Chronic noise exposure, high-frequency hearing loss, and hypertension among automotive assembly workers. J Occup Environ Med. 32:685–689. 2401922

[evz250-B80] Tatusov RL, et al 2003 The COG database: an updated version includes eukaryotes. BMC Bioinformatics 4:41. 12969510 10.1186/1471-2105-4-41 PMC222959

[evz250-B81] Teitelbaum SL. 2000 Bone resorption by osteoclasts. Science 289(5484):1504–1508. 10968780 10.1126/science.289.5484.1504

[evz250-B82] Teitelbaum SL, Ross FP. 2003 Genetic regulation of osteoclast development and function. Nat Rev Genet. 4(8):638. 12897775 10.1038/nrg1122

[evz250-B83] Thomas GWC, Hahn MW. 2015 Determining the null model for detecting adaptive convergence from genomic data: a case study using echolocating mammals. Mol Biol Evol. 32(5):1232–1236. 25631926 10.1093/molbev/msv013 PMC4408409

[evz250-B84] Tong C, Fei T, Zhang C, Zhao K. 2017 Comprehensive transcriptomic analysis of Tibetan Schizothoracinae fish *Gymnocypris przewalskii* reveals how it adapts to a high altitude aquatic life. BMC Evol Biol. 17(1):74. 28274203 10.1186/s12862-017-0925-z PMC5343388

[evz250-B85] van de Kamp JM, et al 2015 Genotype–phenotype correlation of contiguous gene deletions of *SLC6A8, BCAP31* and *ABCD1*. Clin Genet. 87(2):141–147. 24597975 10.1111/cge.12355

[evz250-B86] Wangemann P, Marcus DC. 2017 Ion and fluid homeostasis in the cochlea In: Manly GA, Gummer AW, Popper AN, Fay RR, editors. Understanding the cochlea. Cham: Springer p. 253–286.

[evz250-B88] Yamasoba T, Nuttall AL, Harris C, Raphael Y, Miller JM. 1998 Role of glutathione in protection against noise-induced hearing loss. Brain Res. 784(1–2):82–90. 9518561 10.1016/s0006-8993(97)01156-6

[evz250-B89] Yang CH, Schrepfer T, Schacht J. 2015 Age-related hearing impairment and the triad of acquired hearing loss. Front Cell Neurosci. 9:276. 26283913 10.3389/fncel.2015.00276 PMC4515558

[evz250-B90] Yang S, et al 2016 Immune defense is the primary function associated with the differentially expressed genes in the cochlea following acoustic trauma. Hearing Res. 333:283–294. 10.1016/j.heares.2015.10.010 PMC4798880 26520584

[evz250-B91] Yang Z. 2007 PAML 4: phylogenetic analysis by maximum likelihood. Mol Biol Evol. 24(8):1586–1591. 17483113 10.1093/molbev/msm088

[evz250-B92] Yang Z, Wong WS, Nielsen R. 2005 Bayes empirical bayes inference of amino acid sites under positive selection. Mol Biol Evol. 22(4):1107–1118. 15689528 10.1093/molbev/msi097

[evz250-B94] Ye J, et al 2006 WEGO: a web tool for plotting GO annotations. Nucleic Acids Res. 34(Web Server):W293–W297. 16845012 10.1093/nar/gkl031 PMC1538768

[evz250-B95] Yim HS, et al 2014 Minke whale genome and aquatic adaptation in cetaceans. Nat Genet. 46(1):88. 24270359 10.1038/ng.2835 PMC4079537

[evz250-B96] Zhang J, Kumar S. 1997 Detection of convergent and parallel evolution at the amino acid sequence level. Mol Biol Evol. 14(5):527–536. 9159930 10.1093/oxfordjournals.molbev.a025789

[evz250-B97] Zine A, van de Water TR. 2004 The MAPK/JNK signalling pathway offers potential therapeutic targets for the prevention of acquired deafness. Curr Drug Targets CNS Neurol Disord. 3(4):325–332. 15379608 10.2174/1568007043337166

[evz250-B98] Zou Z, Zhang J. 2015 No genome-wide protein sequence convergence for echolocation. Mol Biol Evol. 32(5):1237–1241. 25631925 10.1093/molbev/msv014 PMC4408410

