# Supplementary material for: Evolutionary Basis of High-Frequency Hearing in the Cochleae of Echolocators Revealed by Comparative Genomics
Source: Genome Biol Evol. 2019 Nov 15;12(1):3740–53. doi: 10.1093/gbe/evz250 (PMC7145703; doi:10.1093/gbe/evz250)
Supplement: evz250_Supplementary_Data [file evz250_supplementary_data.zip › Figure S2.pdf]

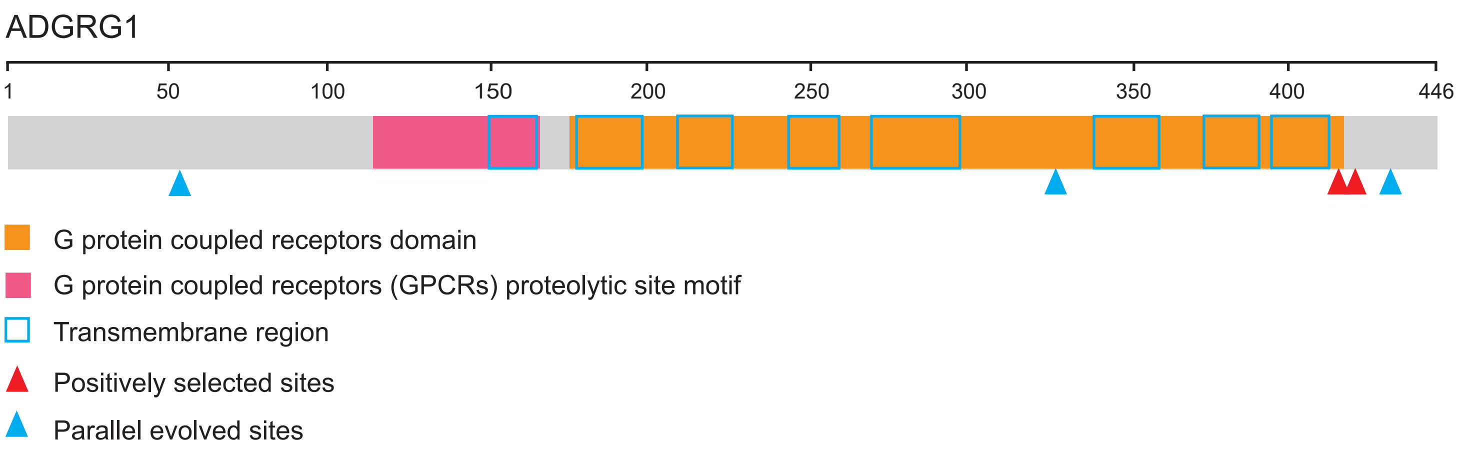
ADGRG1
1
50
100
150
200
250
300
350
400
446
G protein coupled receptors domain
G protein coupled receptors (GPCRs) proteolytic site motif
Transmembrane region
Positively selected sites
Parallel evolved sites

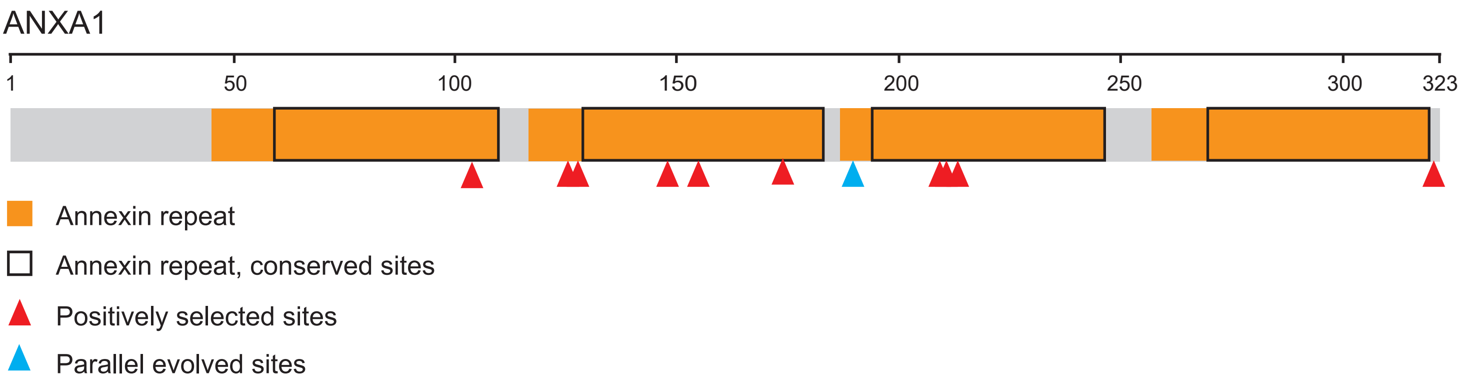
ANXA1
1
50
100
150
200
250
300
323
Annexin repeat
Annexin repeat, conserved sites
Positively selected sites
Parallel evolved sites

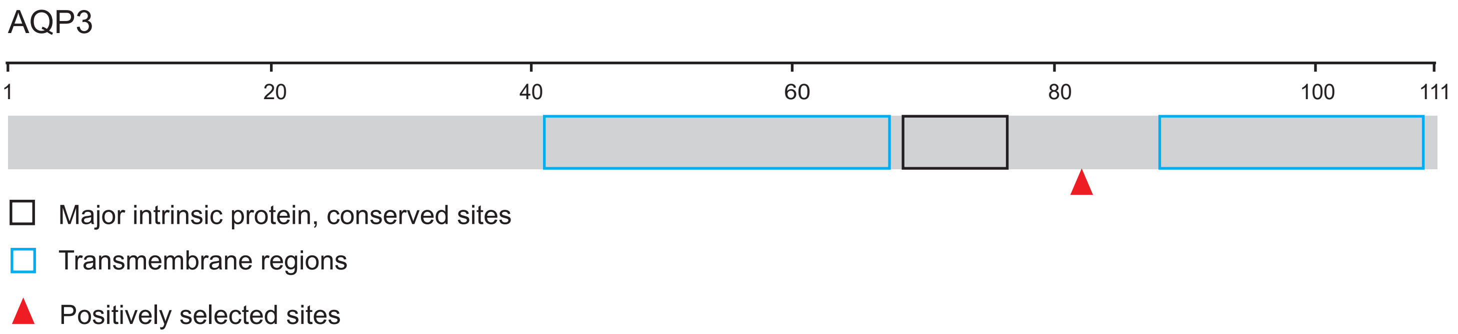
AQP3
1
20
40
60
80
100
111
Major intrinsic protein, conserved sites
Transmembrane regions
Positively selected sites

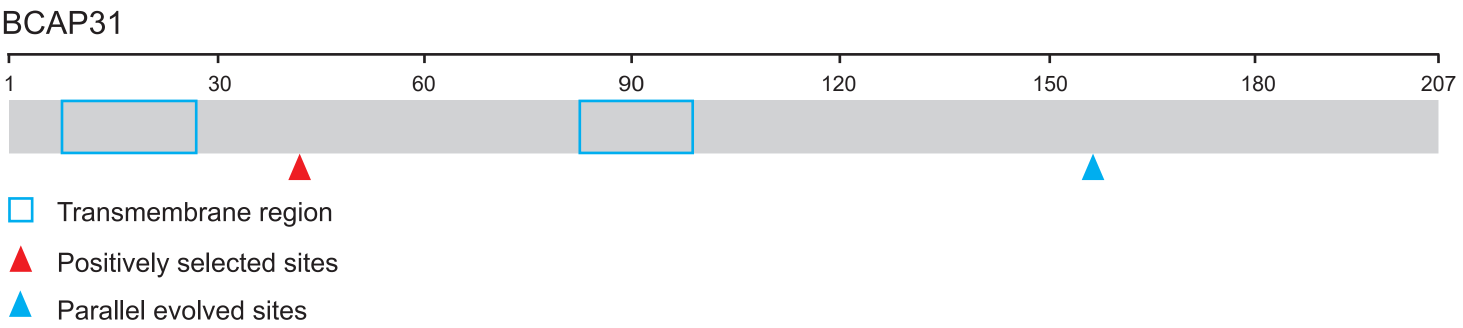
BCAP31
1
30
60
90
120
150
180
207
Transmembrane region
Positively selected sites
Parallel evolved sites

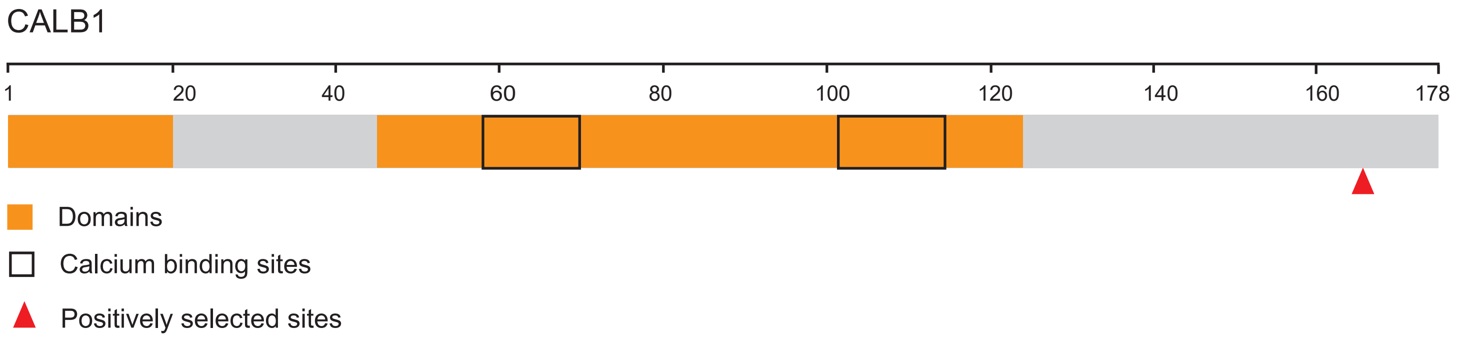
CALB1
1
20
40
60
80
100
120
140
160
178
Domains
Calcium binding sites
Positively selected sites

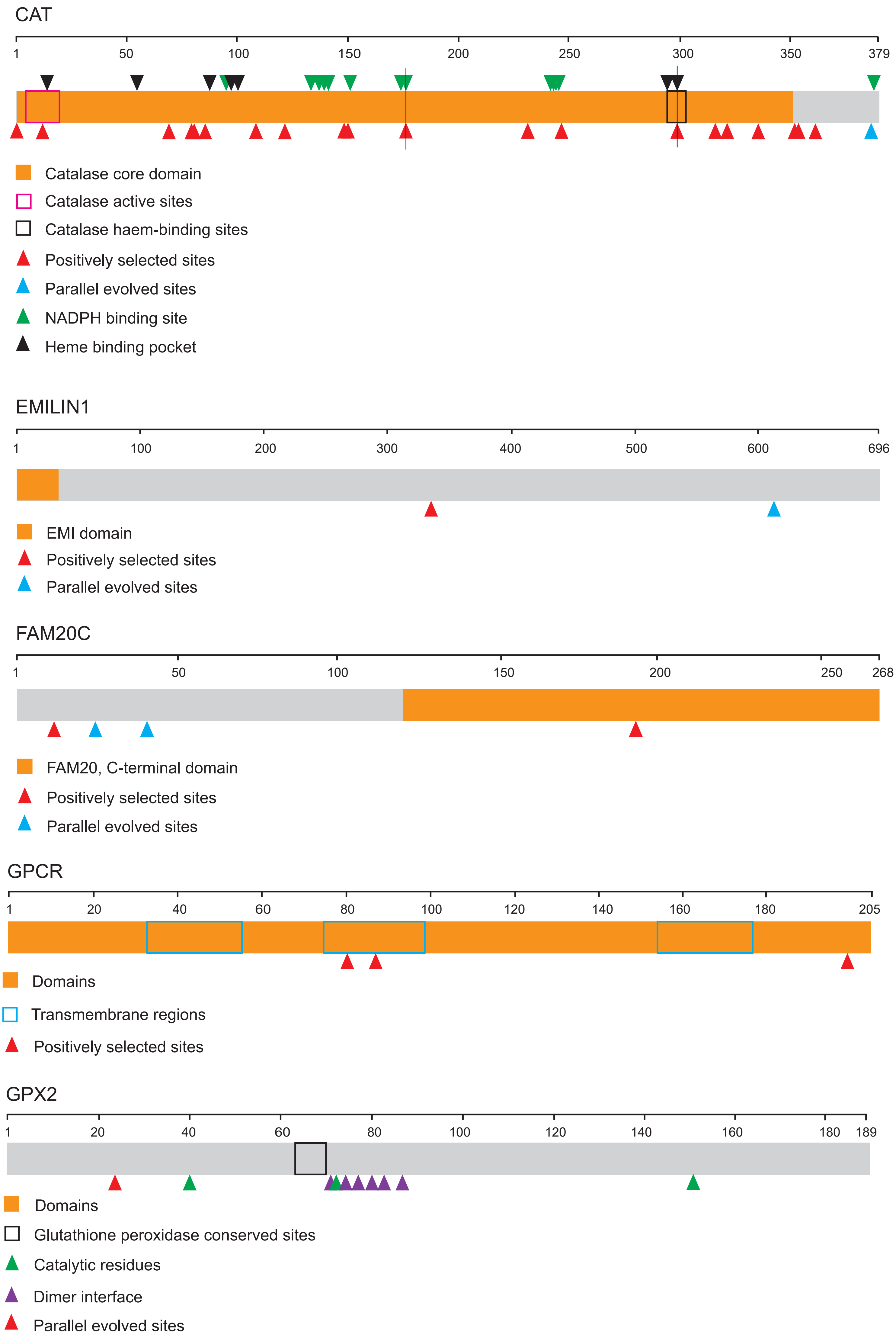
CAT
1 50 100 150 200 250 300 350 379
Catalase core domain
Catalase active sites
Catalase haem-binding sites
Positively selected sites
Parallel evolved sites
NADPH binding site
Heme binding pocket
EMILIN1
1 100 200 300 400 500 600 696
EMI domain
Positively selected sites
Parallel evolved sites
FAM20C
1 50 100 150 200 250 268
FAM20, C-terminal domain
Positively selected sites
Parallel evolved sites
GPCR
1 20 40 60 80 100 120 140 160 180 205
Domains
Transmembrane regions
Positively selected sites
GPX2
1 20 40 60 80 100 120 140 160 180 189
Domains
Glutathione peroxidase conserved sites
Catalytic residues
Dimer interface
Parallel evolved sites

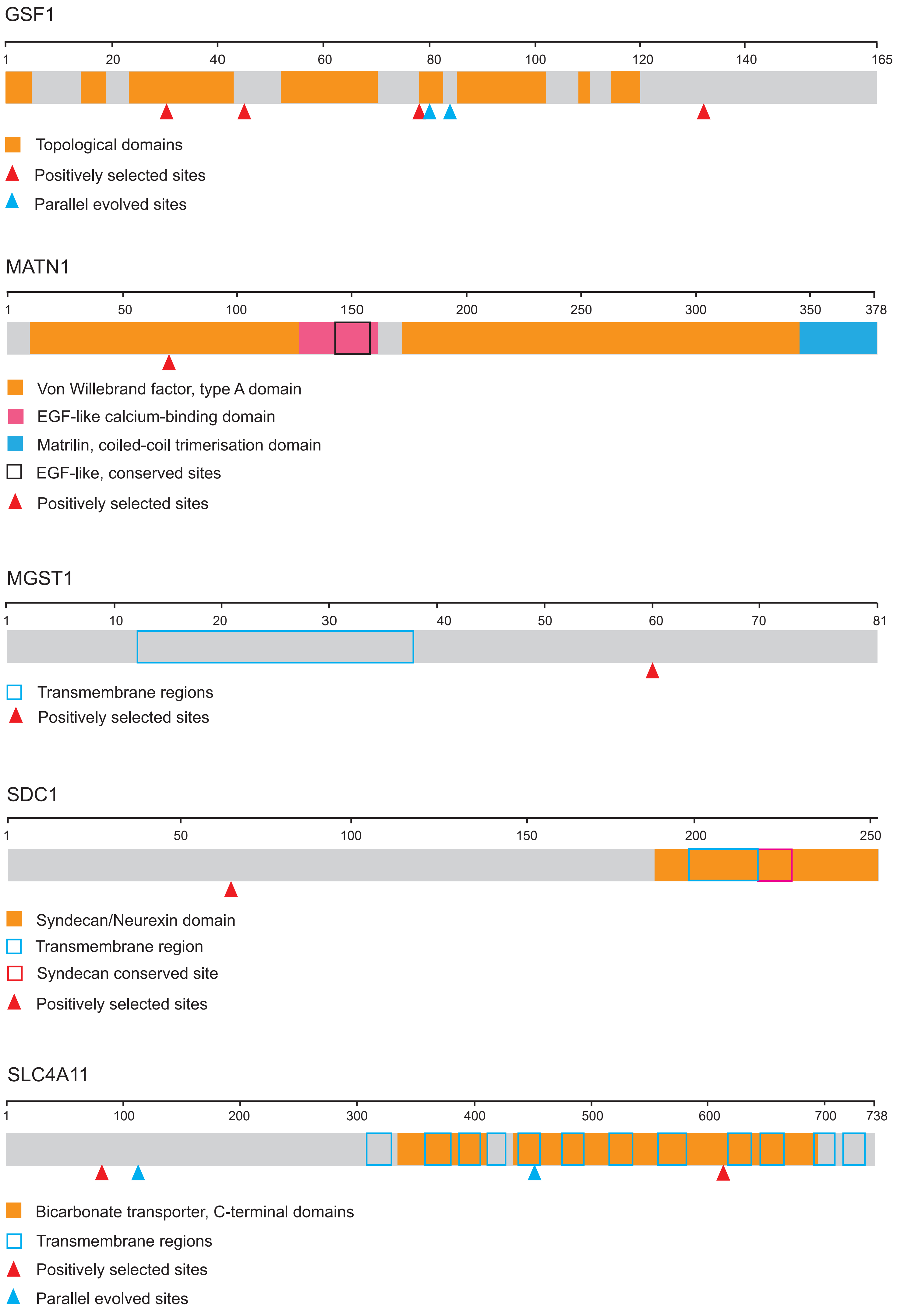
GSF1
1
20
40
60
80
100
120
140
165
Topological domains
Positively selected sites
Parallel evolved sites
MATN1
1
50
100
150
200
250
300
350
378
Von Willebrand factor, type A domain
EGF-like calcium-binding domain
Matrilin, coiled-coil trimerisation domain
EGF-like, conserved sites
Positively selected sites
MGST1
1
10
20
30
40
50
60
70
81
Transmembrane regions
Positively selected sites
SDC1
1
50
100
150
200
250
Syndecan/Neurexin domain
Transmembrane region
Syndecan conserved site
Positively selected sites
SLC4A11
1
100
200
300
400
500
600
700
738
Bicarbonate transporter, C-terminal domains
Transmembrane regions
Positively selected sites
Parallel evolved sites

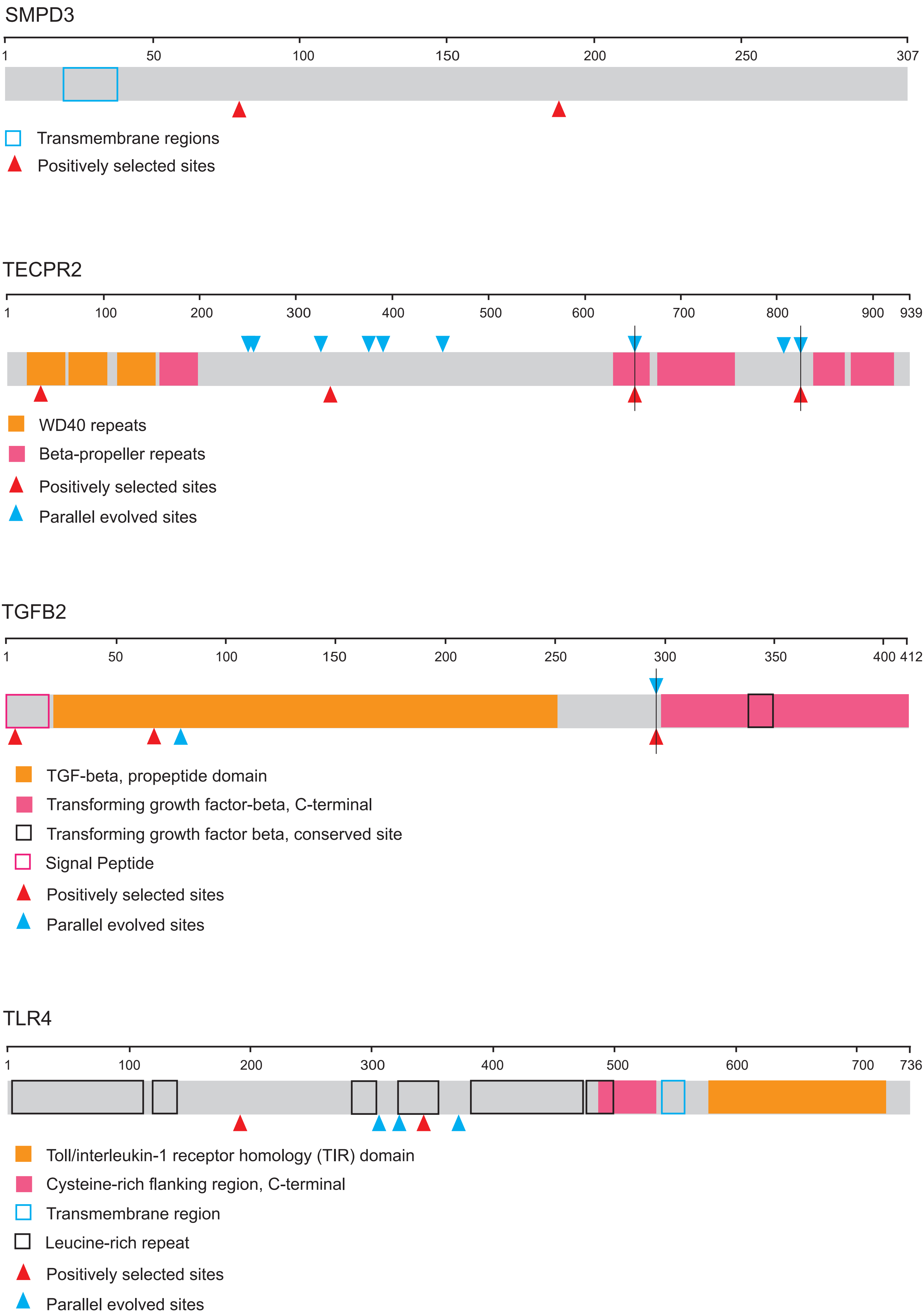
SMPD3
1
50
100
150
200
250
307
Transmembrane regions
Positively selected sites
TECPR2
1
100
200
300
400
500
600
700
800
900
939
WD40 repeats
Beta-propeller repeats
Positively selected sites
Parallel evolved sites
TGFB2
1
50
100
150
200
250
300
350
400 412
TGF-beta, propeptide domain
Transforming growth factor-beta, C-terminal
Transforming growth factor beta, conserved site
Signal Peptide
Positively selected sites
Parallel evolved sites
TLR4
1
100
200
300
400
500
600
700
736
Toll/interleukin-1 receptor homology (TIR) domain
Cysteine-rich flanking region, C-terminal
Transmembrane region
Leucine-rich repeat
Positively selected sites
Parallel evolved sites

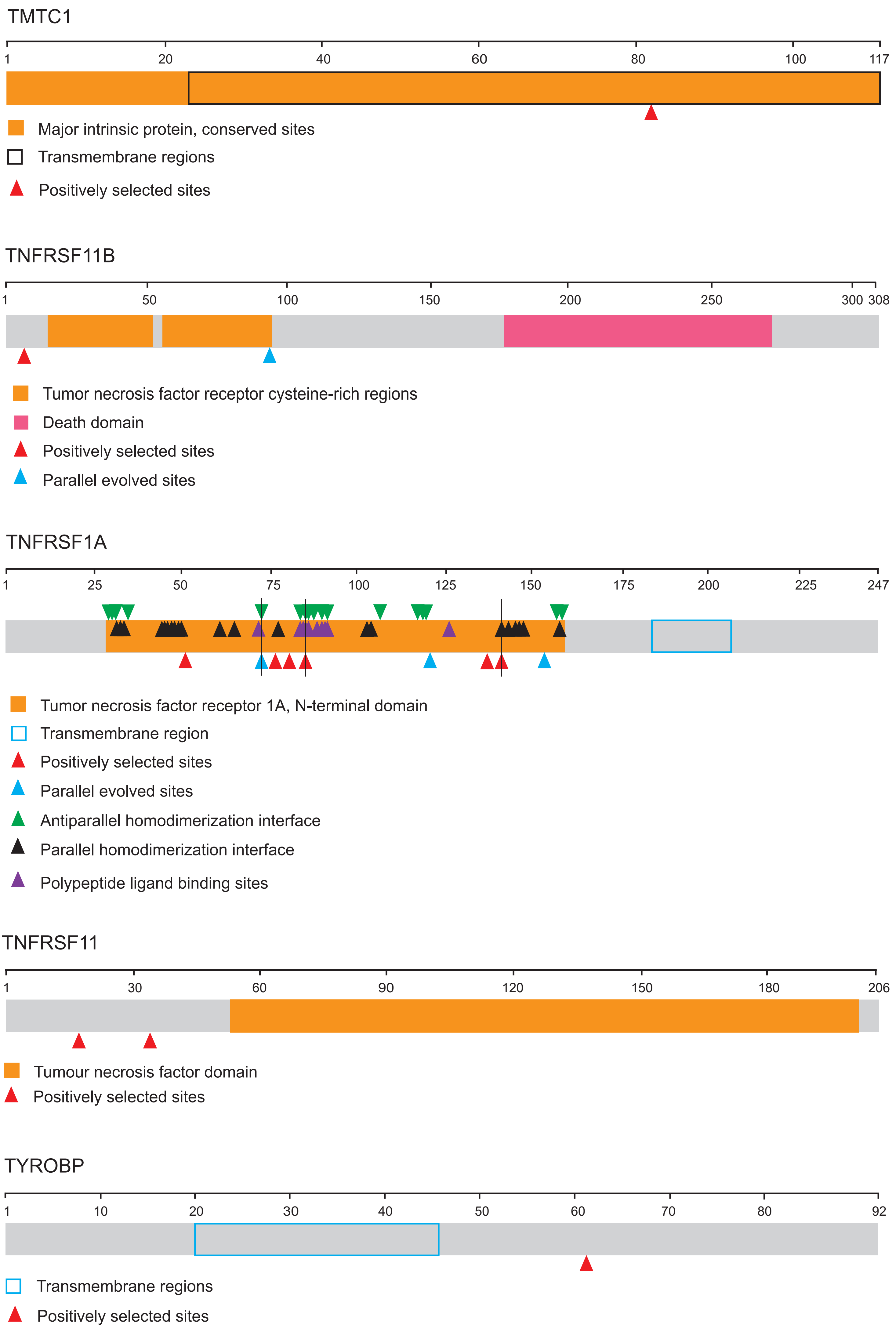

TMTC1
1
20
40
60
80
100
117
Major intrinsic protein, conserved sites
Transmembrane regions
Positively selected sites
TNFRSF11B
1
50
100
150
200
250
300
308
Tumor necrosis factor receptor cysteine-rich regions
Death domain
Positively selected sites
Parallel evolved sites
TNFRSF1A
1
25
50
75
100
125
150
175
200
225
247
Tumor necrosis factor receptor 1A, N-terminal domain
Transmembrane region
Positively selected sites
Parallel evolved sites
Antiparallel homodimerization interface
Parallel homodimerization interface
Polypeptide ligand binding sites
TNFRSF11
1
30
60
90
120
150
180
206
Tumour necrosis factor domain
Positively selected sites
TYROBP
1
10
20
30
40
50
60
70
80
92
Transmembrane regions
Positively selected sites
