# Supplementary material for: Evolutionary Basis of High-Frequency Hearing in the Cochleae of Echolocators Revealed by Comparative Genomics
Source: Genome Biol Evol. 2019 Nov 15;12(1):3740–53. doi: 10.1093/gbe/evz250 (PMC7145703; doi:10.1093/gbe/evz250)
Supplement: evz250_Supplementary_Data [file evz250_supplementary_data.zip › Figure S3.pdf]

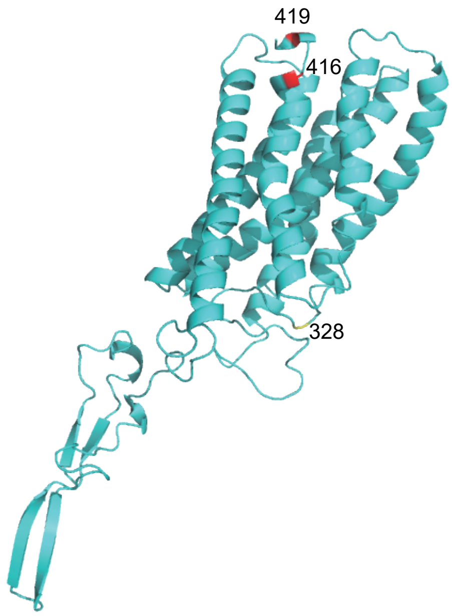

ADGRG1

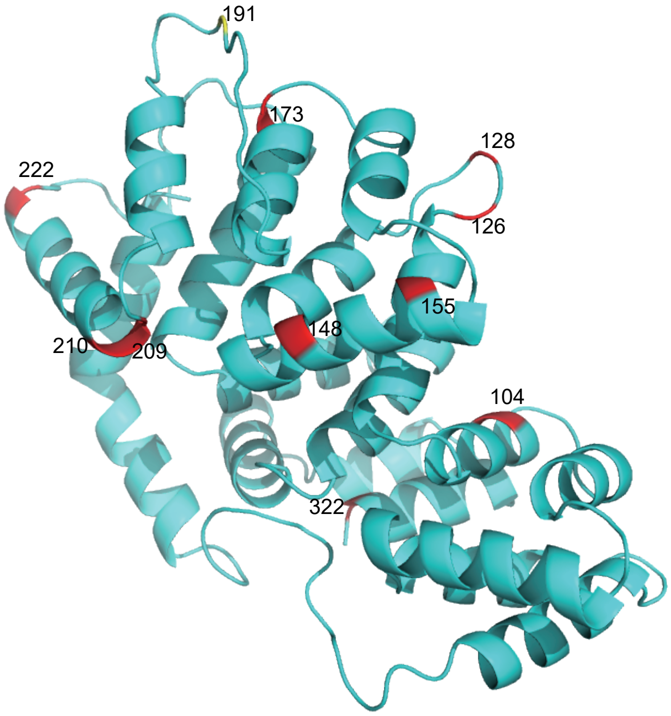

ANXA1

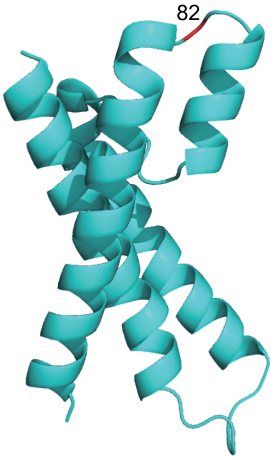

AQP3

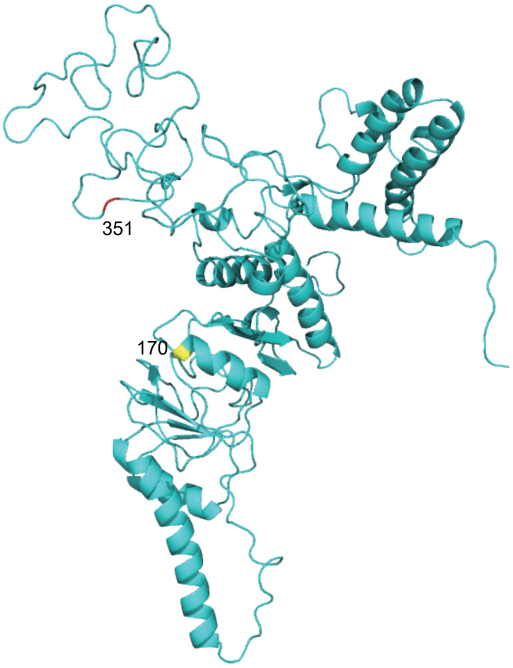

BBS10

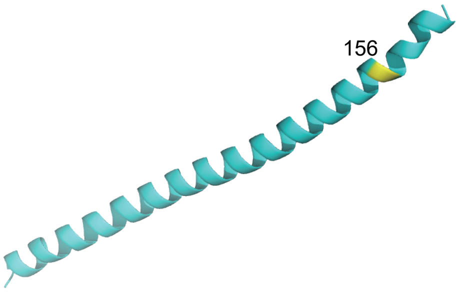

BCAP31

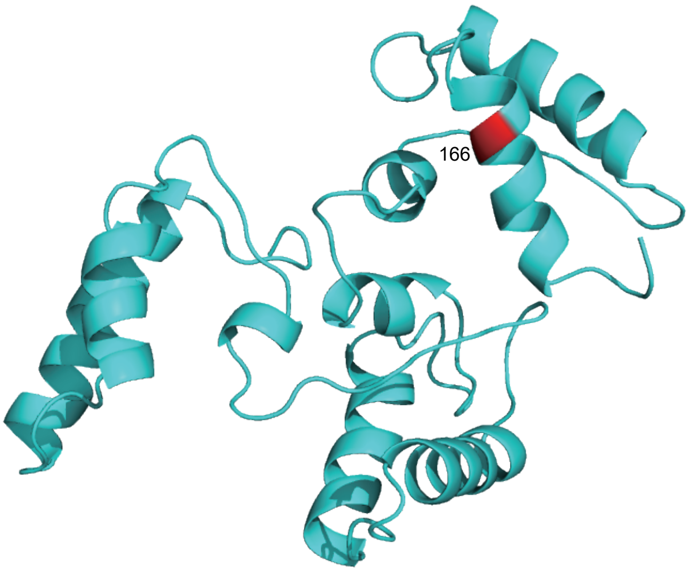

CALB1

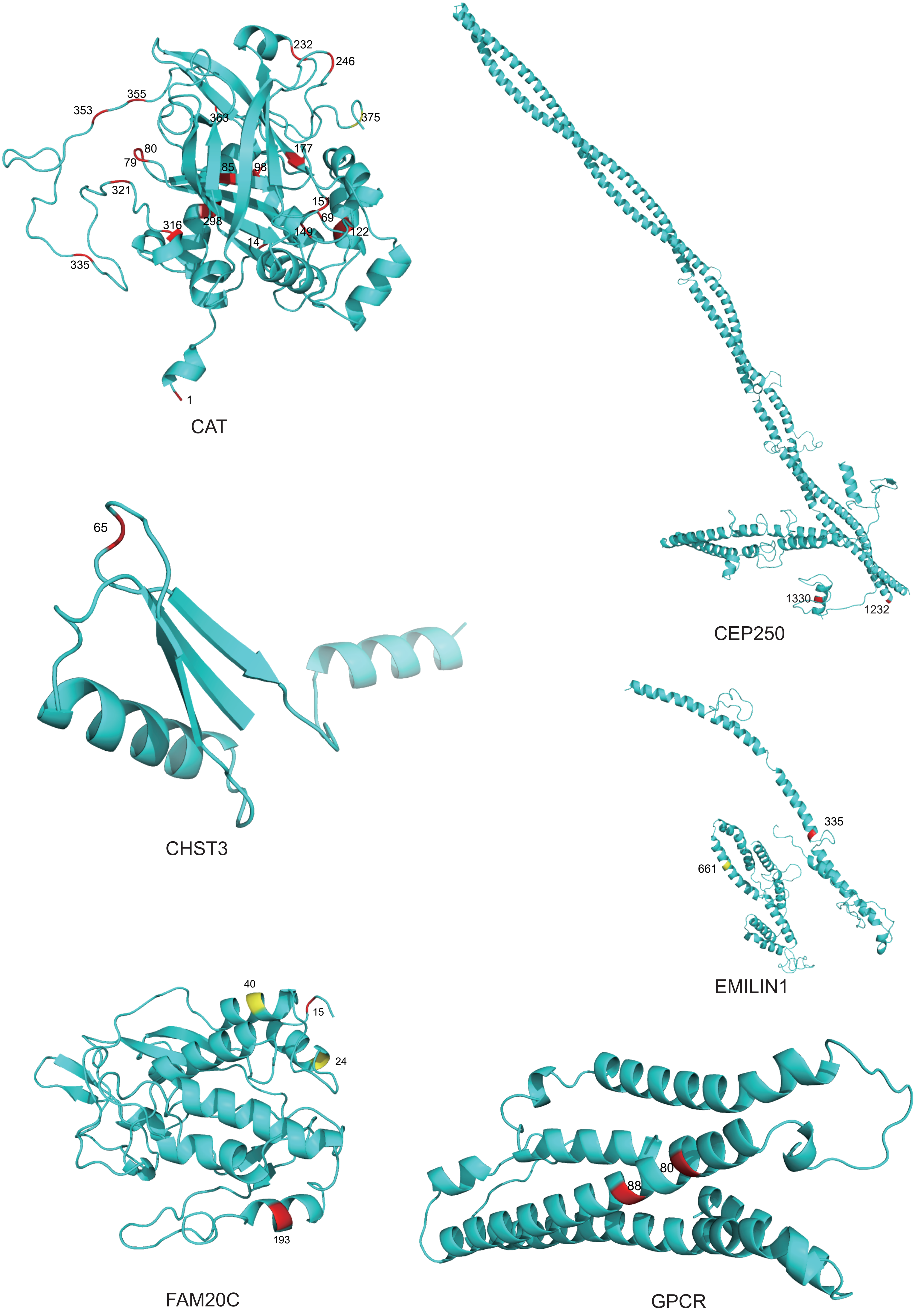
232
246
355
353
363
375
80
79
177
85
98
321
151
69
316
298
149
122
14
335
1
CAT
1330
1232
CEP250
65
CHST3
335
661
EMILIN1
40
15
24
193
FAM20C
80
88
GPCR

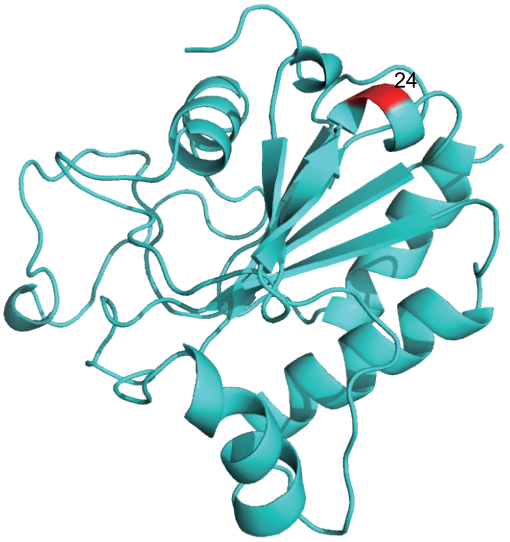

GPX2

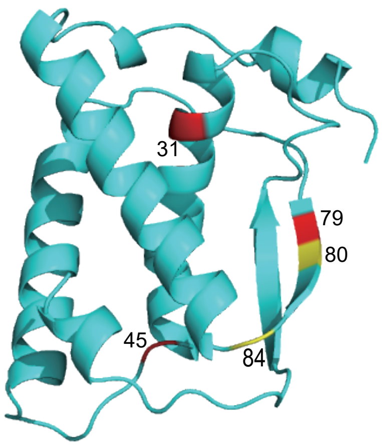

GSF1

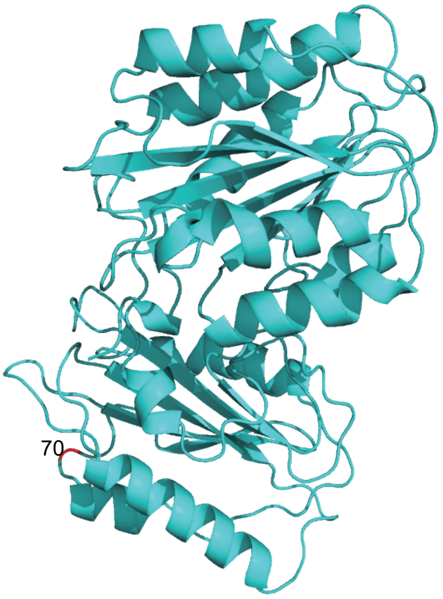

MATN1

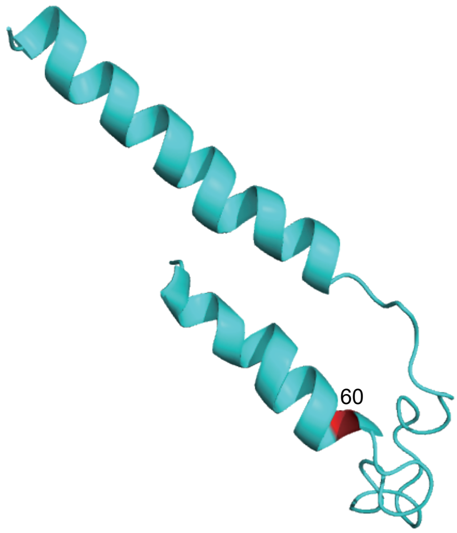

MGST1

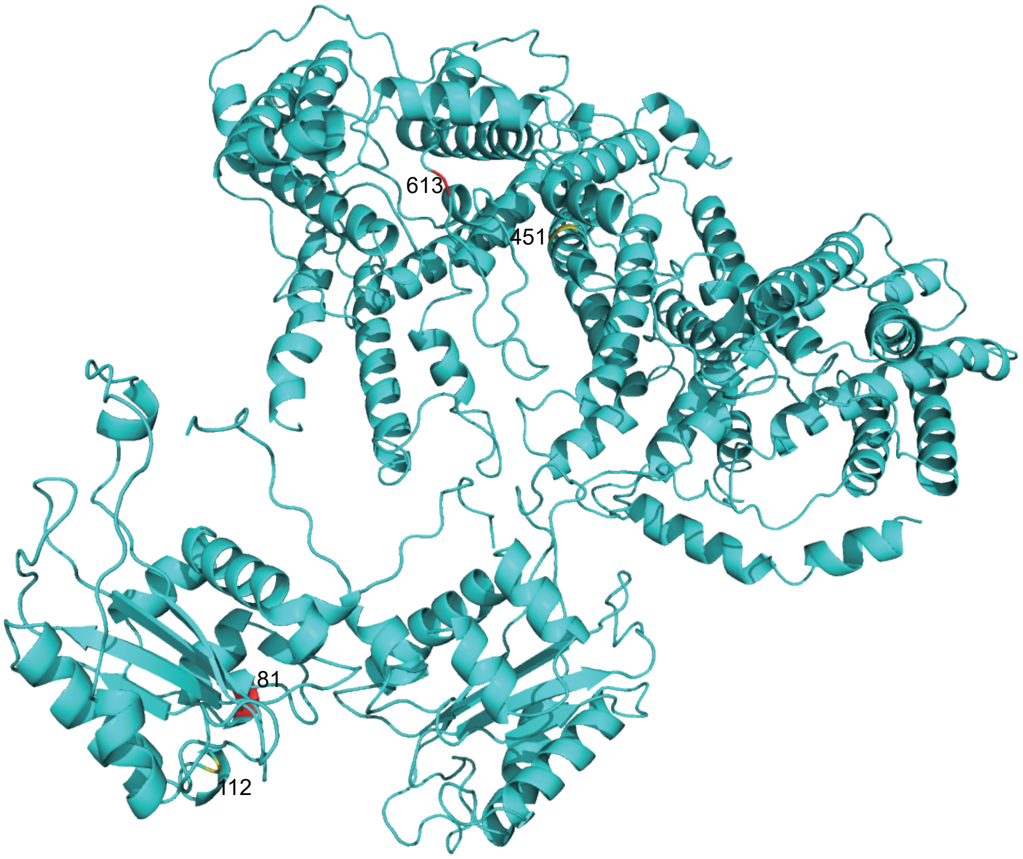

SLC4A11

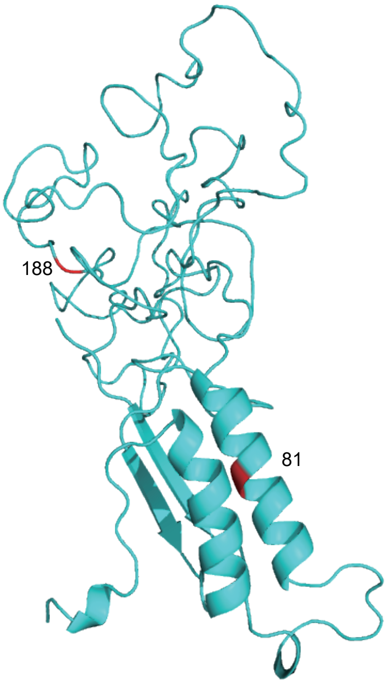

SMPD3

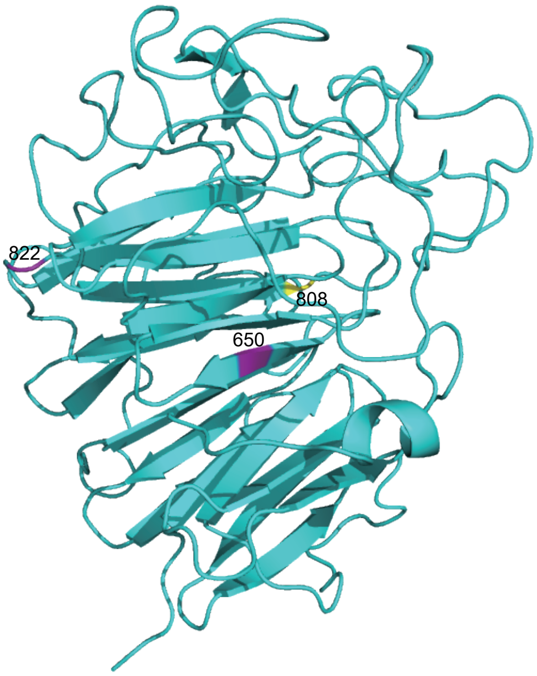

TECPR2

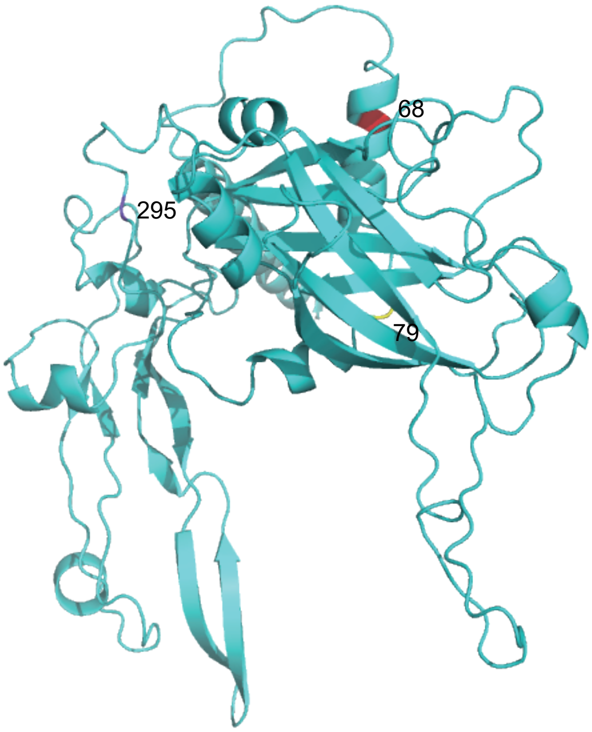

TGFB2

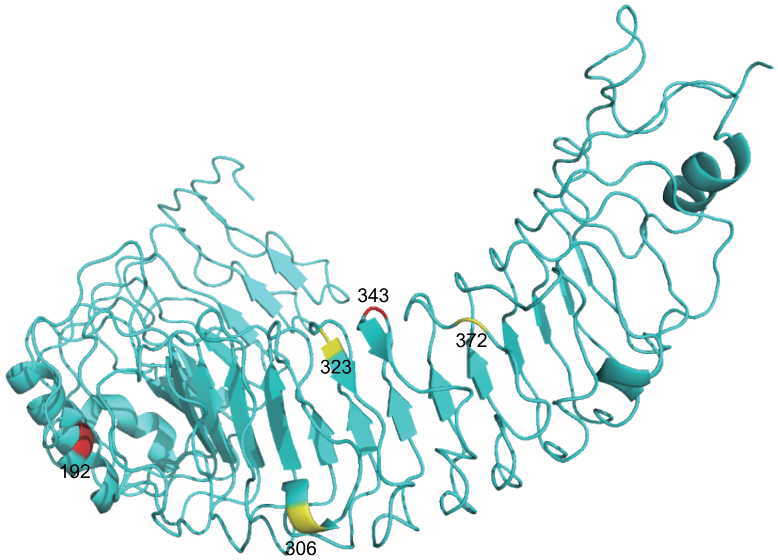

TLR4

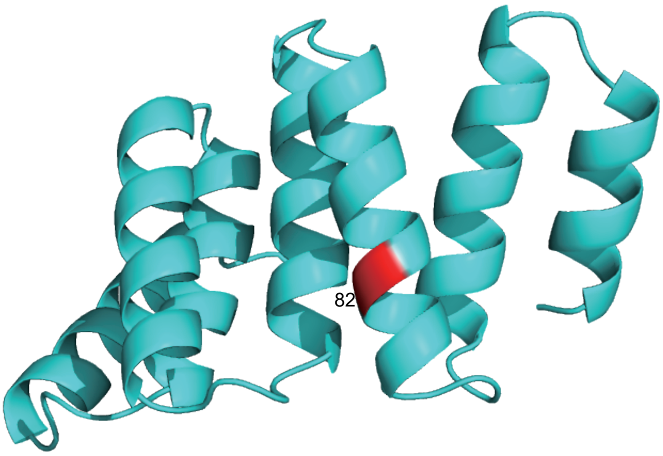

TMTC1

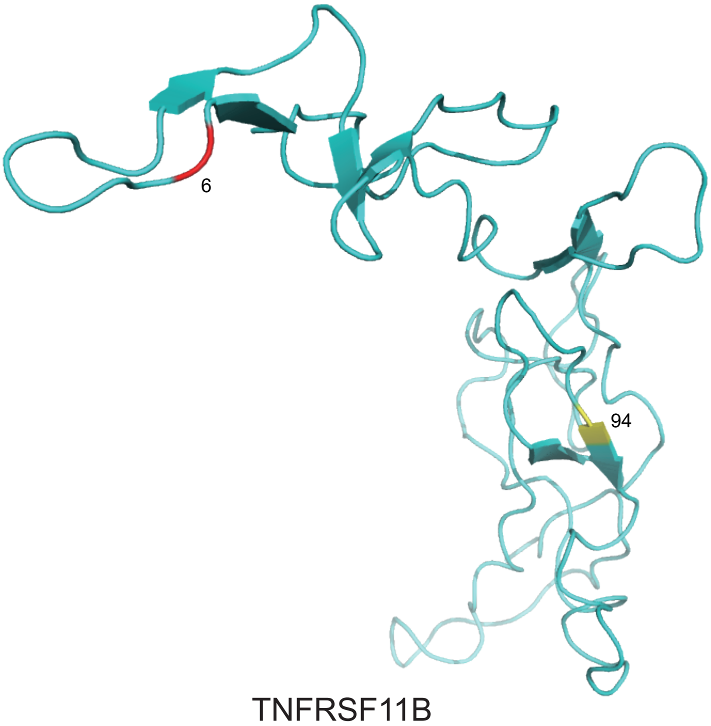

TNFRSF11B

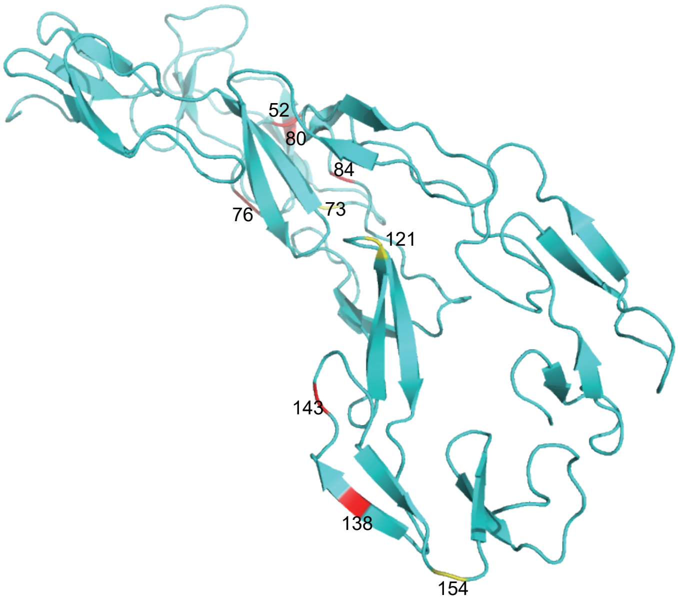

TNFRSF1A

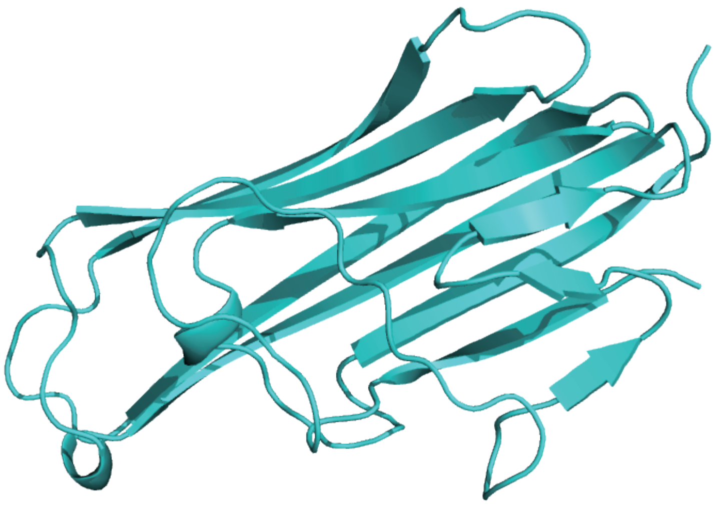

TNFSF11

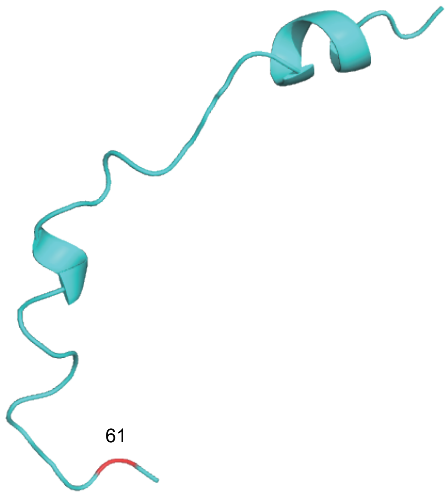

TYROBP
