# Supplementary material for: Evolutionary Basis of High-Frequency Hearing in the Cochleae of Echolocators Revealed by Comparative Genomics
Source: Genome Biol Evol. 2019 Nov 15;12(1):3740–53. doi: 10.1093/gbe/evz250 (PMC7145703; doi:10.1093/gbe/evz250)
Supplement: evz250_Supplementary_Data [file evz250_supplementary_data.zip › Suppl legends proof.docx]

**Supplementary Materials**

**Table S1** Sequencing, assembly, and annotation statistics of *A. stoliczkanus*, *T. melanopogon*, and *R. leschenaultii*, respectively.

**Table S2** Detailed information of selective pressure analyses for 144 PSGs and corresponding positively selected sites along seven foreground branches.

**Table S3** Parallel and convergent genes and sites among six pairwise compared groups. Information of 45 core parallel genes among CF bat, FM bat, click bat, and echolocating toothed whale is also listed in this table.

**Table S4** List of hearing-related PSGs with parallel sites.

**Table S5** GO terms and KEGG pathways significantly associated with 34 hearing-related PSGs and separate hearing-related PSGs along different foreground branches.

**Fig. S1 Various pairwise comparisons of the parallel/convergent analyses. Red lines on the right represent comparisons between echolocating species vs. echolocating species and the blue lines on the left represent comparisons between echolocating species vs. nonecholocating species.**

**Fig. S2** (PDF format) Secondary structure and distribution of positively selected sites and parallel sites in relation to the functionally important regions for 34 hearing-related PSGs.

**Fig. S3** (PDF format) Distribution of positively selected sites (red) and parallel sites (yellow) in the three-dimensional structure of 34 hearing-related PSGs. Most of the protein models used here were mammalian.

**Fig. S4** Protein network analysis of PSGs and parallel genes in the osteoclast differentiation pathway (ko04380). Pink, blue, and gray circles indicate PSGs, parallel genes, and genes under positive selection as well as with parallel sites, respectively. The connecting lines between protein nodes indicate protein–protein interactions.

**Fig. S5** Protein network analysis of PSGs and parallel genes in the NF-κB signaling (ko04064) pathway. Other information in this figure is similar to that in Fig. S4.

General scripts used in this study can be acquired from https://github.com/Huiwanghuiwang/bin and the Perl script for the extraction of one-to-one single-copy orthologs can be found at https://github.com/Huiwanghuiwang/bin/blob/master/orthomcl_findSingleCopyOrtholog.pl.
