# Supplementary figures and images for: Evolutionary Basis of High-Frequency Hearing in the Cochleae of Echolocators Revealed by Comparative Genomics
Source: Genome Biol Evol. 2019 Nov 15;12(1):3740–53. doi: 10.1093/gbe/evz250 (PMC7145703; doi:10.1093/gbe/evz250)

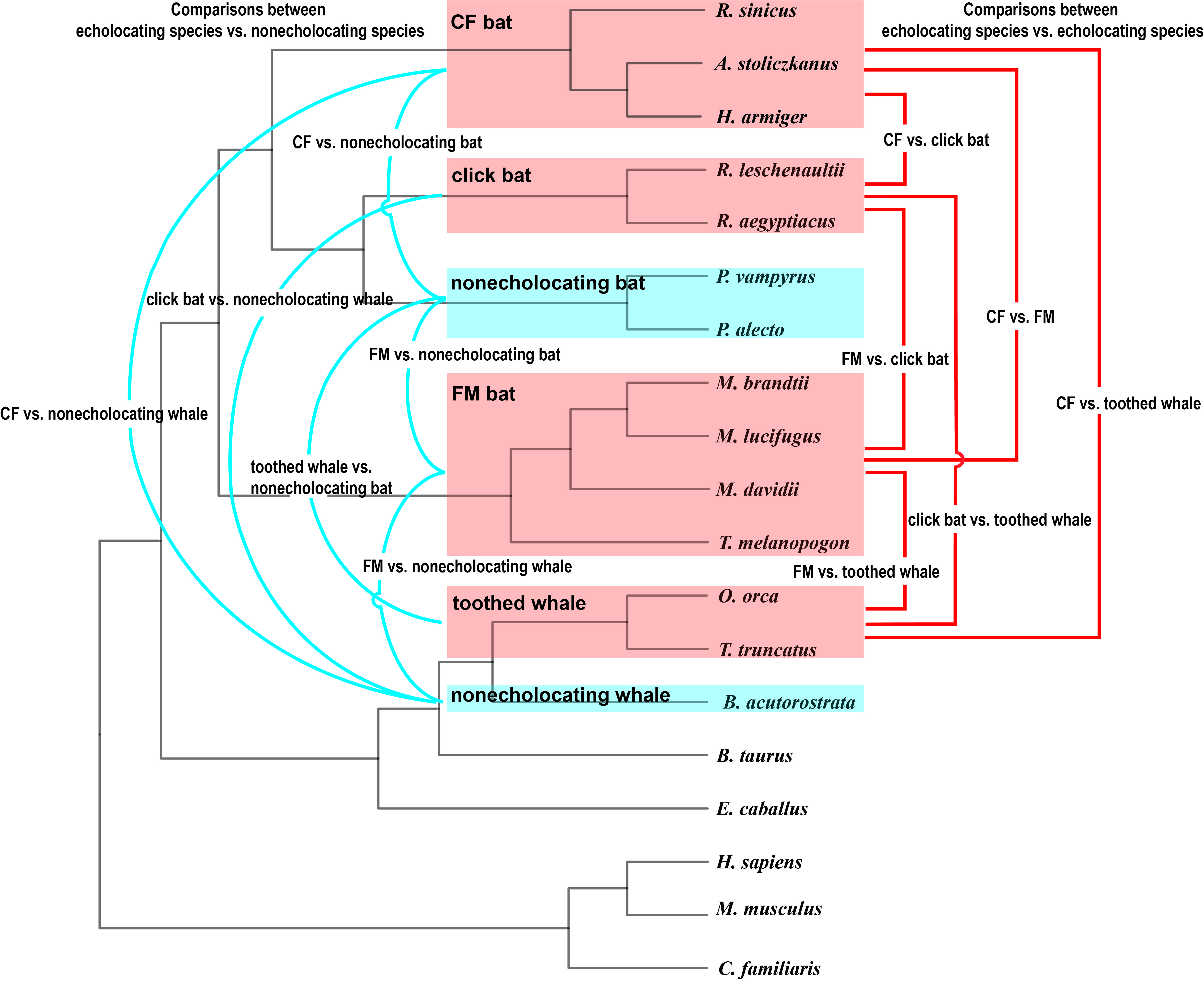

Supplement: evz250_Supplementary_Data [file evz250_supplementary_data.zip › Figure S1.jpg]

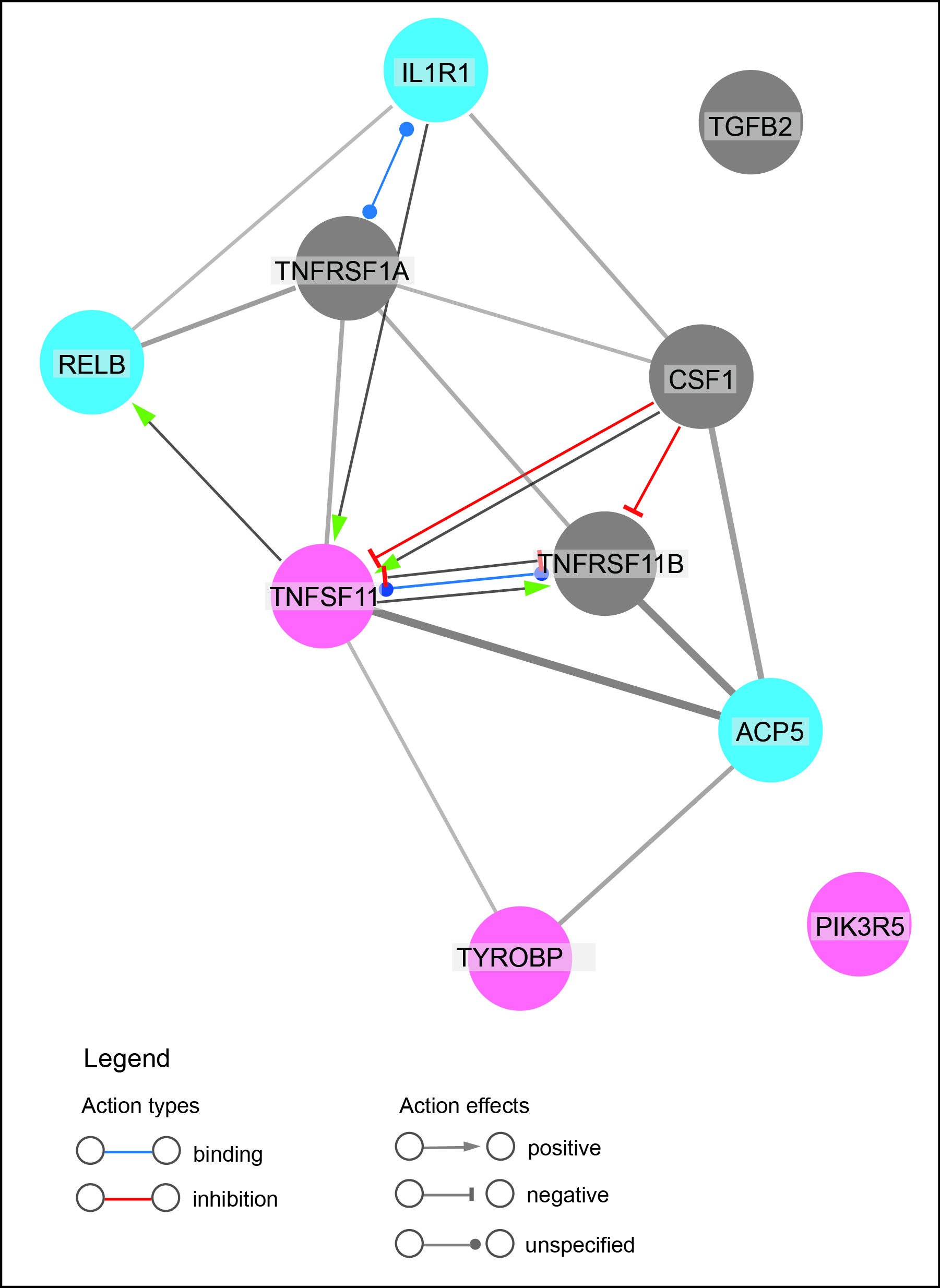

Supplement: evz250_Supplementary_Data [file evz250_supplementary_data.zip › Figure S4.jpg]

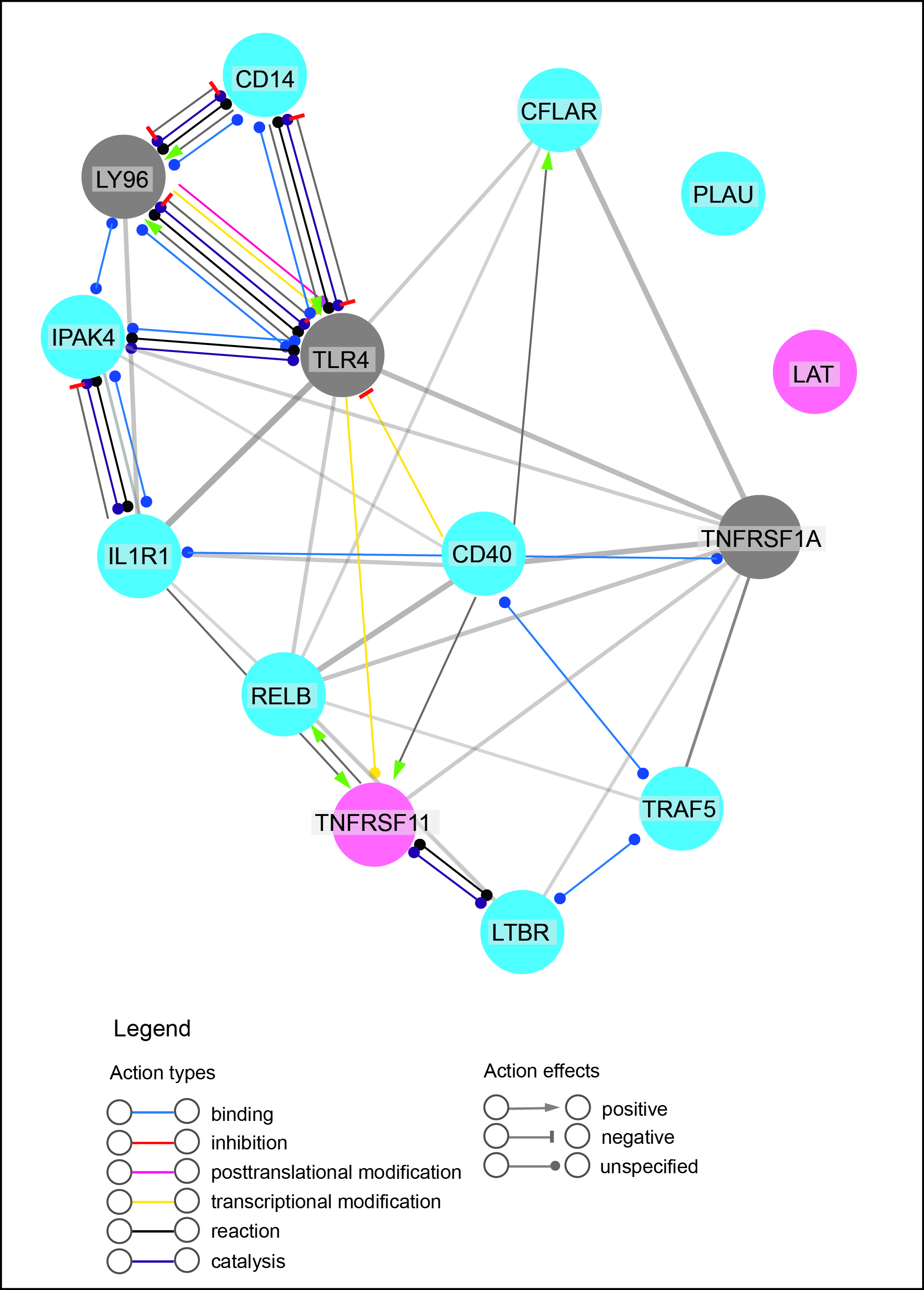

Supplement: evz250_Supplementary_Data [file evz250_supplementary_data.zip › Figure S5.jpg]
